# miR-802 regulates Paneth cell function and enterocyte differentiation in the mouse small intestine

**DOI:** 10.1038/s41467-021-23298-3

**Published:** 2021-06-07

**Authors:** Algera Goga, Büsra Yagabasan, Karolin Herrmanns, Svenja Godbersen, Pamuditha N. Silva, Remy Denzler, Mirjam Zünd, Markus Furter, Gerald Schwank, Shinichi Sunagawa, Wolf-Dietrich Hardt, Markus Stoffel

**Affiliations:** 1 Institute of Molecular Health Sciences, ETH Zurich, Zürich, Switzerland; 2 Laboratory of Microbiome Research, Institute of Microbiology and Swiss Institute of Bioinformatics, ETH Zürich, Zürich, Switzerland; 3 Institute of Microbiology, ETH Zurich, Zürich, Switzerland; 4 Institut für Pharmakologie und Toxikologie, Universität Zürich, Zürich, Switzerland; 5 Medical Faculty, University of Zürich, Zürich, Switzerland; 6 Present Address: Section on Vascular Cell Biology, Joslin Diabetes Center and Department of Medicine, Harvard Medical School, Boston, MA USA; 7 Present Address: Astra Zeneka, Section Oncology & Hematology, Baar, Zug, Switzerland

**Keywords:** Gene expression, miRNAs

## Abstract

The intestinal epithelium is a complex structure that integrates digestive, immunological, neuroendocrine, and regenerative functions. Epithelial homeostasis is maintained by a coordinated cross-talk of different epithelial cell types. Loss of integrity of the intestinal epithelium plays a key role in inflammatory diseases and gastrointestinal infection. Here we show that the intestine-enriched *miR-802* is a central regulator of intestinal epithelial cell proliferation, Paneth cell function, and enterocyte differentiation. Genetic ablation of *mir-802* in the small intestine of mice leads to decreased glucose uptake, impaired enterocyte differentiation, increased Paneth cell function and intestinal epithelial proliferation. These effects are mediated in part through derepression of the *miR-802* target *Tmed9*, a modulator of Wnt and lysozyme/defensin secretion in Paneth cells, and the downstream Wnt signaling components *Fzd5* and *Tcf4*. Mutant *Tmed9* mice harboring mutations in *miR-802* binding sites partially recapitulate the augmented Paneth cell function of mice lacking *miR-802*. Our study demonstrates a broad *miR-802* network that is important for the integration of signaling pathways of different cell types controlling epithelial homeostasis in the small intestine.

## Abstract (web summary)

Homeostasis of the intestinal epithelium is crucial for the maintenance of the epithelial barrier. Here, the authors show that *mir-802* ablation in the mouse intestine impairs enterocyte differentiation and glucose absorption, enhances Paneth cell function by increasing Tmed9-mediated defensin secretion, and increases epithelial cell turnover.

## Introduction

MicroRNAs (miRs) are essential post-transcriptional repressors of mRNA targets influencing complex gene networks that regulate fundamental processes of cell differentiation, proliferation, development, and homeostasis^1,2^. Genetic studies in mice have been crucial in linking miRNA function to developmental, cellular, physiological, and behavioral phenotypes^3^. Importantly, many miRNA knock-out mouse models exhibit differential responses to disease or injury and some have revealed altered susceptibility to infection^4–6^.

miRs are grouped into families based on their targeting properties, which depend primarily on the base pair identity of their extended seed region (i.e., miRNA nucleotides 2–8)^1^. Of the ≈500 canonical miRNA genes that have been identified in the human genome, *miR-802* is a unique member of 62 seed families that are conserved from bony fish to vertebrates^7^. Despite its evolutionary conservation, our biological understanding of *miR-802* function on a cellular and organismic level is scarce. *MicroRNA-802* was first studied in the liver, where it was reported to be upregulated in insulin-resistant and obese states^8^. Other studies have suggested that *miR-802* may play a role in insulin secretion and have anti-oncogenic properties in organs of the GI tract^9–11^. Furthermore, it has been shown that hepatic *miR-802* levels are higher in females than males and that plasma *miR-802* levels are increased in rats when treated with nephron- or hepatotoxicants^12,13^.

Intestines evolved in evolution as early as teleost fish, where they have an important role in increasing both the surface area and the effective length of the intestine for absorption of macro and micronutrients from food^14^. The epithelium of the small intestine is organized into large numbers of self-renewing crypt-villus units. The base of each villus is surrounded by multiple epithelial invaginations, that contain highly proliferating stem cells, which sustain the self-renewal of the epithelium. Six differentiated epithelial cell types are distinguished in the intestine: the absorptive enterocytes, goblet cells, and enteroendocrine cells that secrete mucus and a variety of hormones, respectively, Tuft cells that may sense luminal contents, microfold (M) cells that overlie the Peyer’s patches, and Paneth cells that occupy the bottom positions in the crypt adjacent to intestinal stem cells^15^. Paneth cells secrete bactericidal products such as lysozyme and defensins, play key roles in mucosal immunity, provide niche factors to support crypt base columnar cells (CBC), including epidermal growth factor (EGF), Wnt (WNT3A) and Notch ligands, and bone morphogenetic protein (BMP) inhibitor Noggin^16^.

In this study, we describe an important role of *miR-802* in the maintenance of intestinal homeostasis. We demonstrate that genetic deletion of *miR-802* expression or mutations in *miR-802* binding sites of a single target gene results in distinct phenotypes and leads to dysregulated gene networks influencing epithelial glucose uptake, proliferation, differentiation, and innate immunity.

## Results

### *mir-802* deletion in mice impairs intestinal glucose transport

**Fig. 1:**
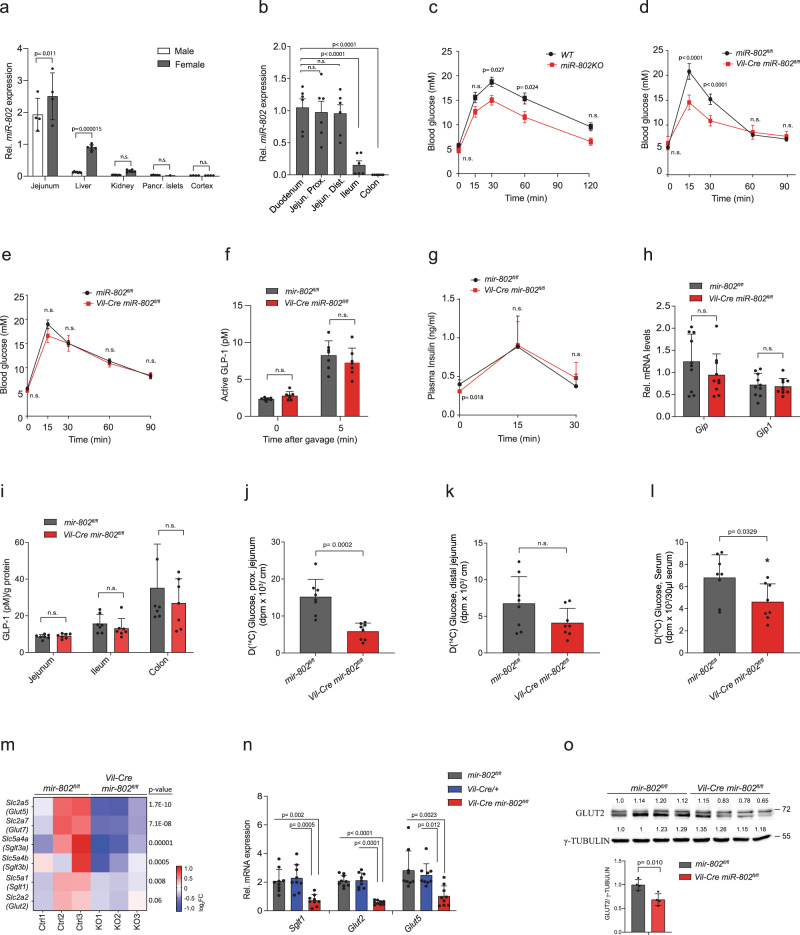
*miR-802* regulates glucose absorption in the small intestine. **a** Relative expression levels of *miR-802* in indicated tissues (jejunum *n* = 4,4; liver *n* = 7,6; kidney *n* = 7,6; pancreatic islet *n* = 5,1; cortex *n* = 4,4). Data are representative of three independent experiments. **b** Relative *miR-802* expression in female mice, measured by qRT-PCR, in indicated intestine sections (*n* = 6). Data are representative of two independent experiments **c** Oral glucose tolerance test (OGTT) of *mir-802KO* and control littermates (WT) (*n* = 8,9 per genotype). **d**, **e** OGTT (**d**) and IPGTT (**e**) in *Vil-Cre mir-802*^*fl/fl*^ and *mir-802*^*fl/fl*^ controls (*n* = 8,6 respectively for (**d**), *n* = 6 for (**e**)). **f**, **g** Serum GLP1 and insulin measurements of *Vil-Cre mir-802*^*fl/fl*^ and control mice at the indicated time points after the oral glucose challenge (for (**f**) *n* = 7 for each genotype, for (**g**): wt *n* = 6 and KO *n* = 9). **h** Relative mRNA levels of *Gip* and *Glp1* in small intestines of *Vil-Cre mir-802*^*fl/fl*^ and control mice (*n* = 10 per group). **i** GLP1 protein measurements of jejunum from Vil-Cre *mir-802*^*fl/fl*^ and control mice (*n* = 7 per group, one experiment). **j**–**l** D-^14^C-Glucose levels in proximal jejunum (**j**), distal jejunum (**k**), and serum (**l**) following an oral ^14^C-glucose gavage (*n* = 8 for all groups, one experiment). **m** Heatmap representation of mRNA expression by RNA seq of glucose transporters in isolated jejunal enterocytes of *Vil-Cre mir-802*^*fl/fl*^ and control mice shown as Log2FC (*n* = 3). **n** Relative mRNA expression, measured by qRT-PCR, of indicated glucose transporters in jejunal enterocytes of *Vil-Cre mir-802*^*fl/fl*^ and indicated control mice (*n* = 9 per genotype). Data are representative of two independent experiments. **o** Immunoblot of GLUT2 in isolated enterocytes of the proximal jejunum of *Vil-Cre mir-802*^*fl/fl*^ and control mice. γ-TUBULIN was used as a loading control. Quantification of densitometric analysis of signals is shown on the right. Each lane represents a lysate from a different mouse (*n* = 4 per genotype, one experiment). Data are plotted as mean ± SD. Significance was evaluated by two-tailed *t* test (**j**–**l**, **o**), by multiple two-tailed *t*-tests with Holm−Sidak method for multiple comparisons (**a**, **h**, **i**), one-way ANOVA with Tukey’s multiple comparison post-test (**n**), or Dunnet post test (**b**), and two-way ANOVA for repeated measures with Sidak’s multiple comparisons test (**c**–**g**).

Expression analysis of *miR-802* levels in multiple tissues revealed that the jejunum had significantly higher levels than previously studied organs (Fig. 1a)^8^. A more detailed analysis of *miR-802* in the entire gut revealed the highest levels in the upper small intestine (duodenum and jejunum), intermediate levels in the ileum and undetectable expression in the colon (Fig. 1b). Expression of *miR-802* in the upper intestine was slightly higher in females than males, a finding that was previously reported in the liver^12^ (Supplementary Fig. 1a). Since miRNA knockout phenotypes are most often observed in organs with high miRNA expression levels, we investigated mainly female mice with a global (referred to as *mir-802KO*) or intestine-specific deletion of *mir-802* (*Vil-Cre mir-802*^*fl/fl*^) (Supplementary Fig. 1b, c). Given the prominent role of the jejunum in nutrient uptake and previous studies showing the involvement of *mir-802* in glucose homeostasis, we first studied the effect of *mir-802* ablation on glucose metabolism. Mice had similar body weight, blood glucose levels and, intraperitoneal glucose and insulin tolerance tests (IPGTT and IPITT, respectively) (Supplementary Fig. 1d–g). However, when mice were given an oral glucose load by gavage (oral glucose tolerance test, OGTT), we observed lower blood glucose levels in *mir-802KO* and *Vil-Cre mir-802*^*fl/fl*^ mice, whereas IPGTT and IPITT were similar in mutant mice compared to littermates control animals (Fig. 1c–e, Supplementary Fig. 1g, h). These data were also confirmed in male mice. These results indicate that the ablation of *mir-802* in the intestinal epithelium may be responsible for this effect by either influencing incretin secretion from enteroendocrine cells of the gut epithelium or by affecting glucose absorption^17^. Measurements of plasma glucagon-like peptide 1 (GLP-1) and insulin after an oral glucose load revealed similar levels in *Vil-Cre mir-802*^*fl/fl*^ and control mice (Fig. 1f, g). Furthermore, expression of glucose-dependent insulinotropic peptide (*GIP*) and *GLP-1* as well as total GLP-1 content in jejunal tissue revealed no change (Fig. 1h, i). Having ruled out an incretin effect as the cause for the improved glucose tolerance, we next hypothesized that the glucose phenotype might be related to altered intestinal glucose absorption. We performed radioactive glucose uptake assays following oral administration of ^14^C-glucose in *Vil-Cre mir-802*^*fl/fl*^ mice and *mir-802*^*fl/fl*^ littermates and measured postprandial ^14^C-glucose levels in the blood and tissues of the proximal and distal jejunum. ^14^C-glucose was markedly lower in the proximal jejunum of *Vil-Cre mir-802*^*fl/fl*^ mice where most monosaccharides are absorbed; moreover, 15 min after gavage, ^14^C-glucose levels in the circulation were reduced in *Vil-Cre mir-802*^*fl/fl*^ mice compared to controls (Fig. 1j–l). We also analyzed the expression of glucose transporters in isolated jejunal enterocytes and found a ≈60% decrease of *Glut2*, *Sglt1*, and *Glut5* transcripts in *Vil-Cre mir-802*^*fl/fl*^ compared to *mir-802*^*fl/fl*^ or *Vil-Cre* control mice (Fig. 1m, n). Western blotting of GLUT2 *Vil-Cre mir-802* ^*fl/fl*^ mice (Fig. 1o). Together, these results demonstrate that *miR-802* influences glucose tolerance by regulating intestinal glucose absorption through downregulation of intestinal glucose transporter expression.

### *miR-802* regulates Paneth cell expansion and function

**Fig. 2:**
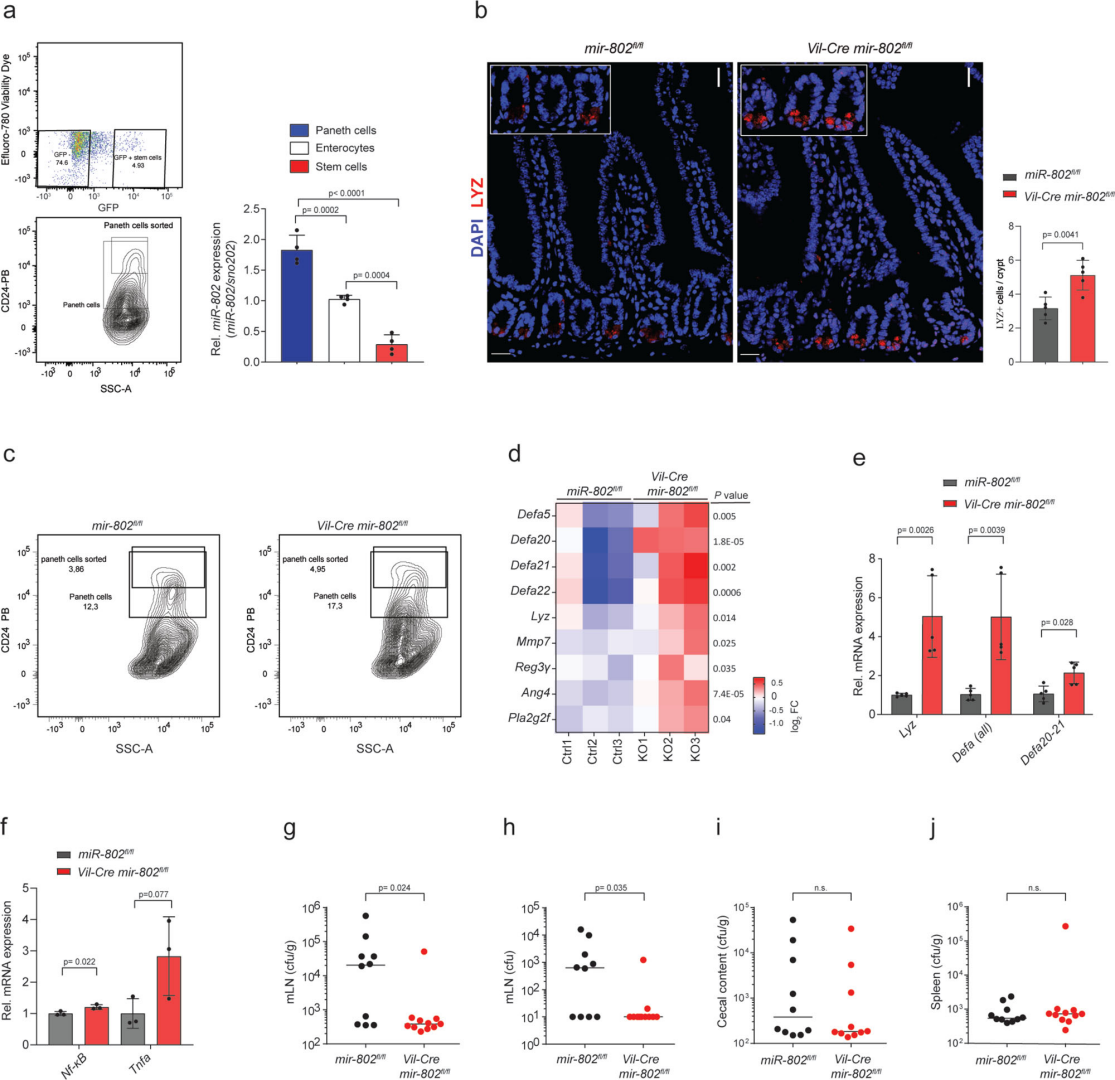
*miR-802* regulates intestinal Paneth cell expansion. **a** FACS sorting of Paneth cells, intestinal stem cells (ISCs), and enterocytes from dissociated single cells of jejunal crypts and epithelium. ISCs were isolated as Lgr5-EGFP^+^, EPCAM^+^, CD24low/CD31^−^, Ter119^−^, CD45^−^, Efluoro780^−^ and Paneth cells were sorted as CD24hi/SSChiLgr5-EGFP^−^, EPCAM^+^CD31^‒^, Ter119^−^, CD45^−^, Efluoro780^−^ cells. An enriched enterocyte population was obtained by isolating remaining CD24^−^ Lgr5-EGFP^−^, EPCAM^+^, CD31^−^, Ter119^−^, CD45^−^, Efluoro780^−^ cells. Right: Relative *miR-802* levels in Paneth cells, enterocytes, and stem cells as determined by qRT-PCR (*n* = 4 per group). Data representative of two independent experiments. **b** Immunohistochemistry of proximal jejunal sections of *mir-802*^*fl/fl*^ and *Vil-Cre mir-802*^*fl/fl*^ mice stained with anti-LYZ antibody. Top panel: Scale bar: 50 µm. Right: Quantification of Paneth cell numbers/crypt (*n* = 5 per genotype). Representative images and analysis of two independent experiments. **c** Percentage of sorted Paneth cells from single live cells from jejunal crypts of *Vil-Cre mir-802*^*fl/fl*^ and control mice. Paneth cells were gated as CD24^+^, SSC^hi^ (*n* = 4 mice were pooled per genotype). Data representative of two independent experiments. **d** Heatmap showing mRNA expression, measured by RNA seq, of secreted proteins in *Vil-Cre mir-802* ^*fl/fl*^ and control mice. Data are shown as Log2FC (*n* = 3). **e**, **f** Relative expression of *Lyz* and *defensins* (**e**) and *NF-κB* and *TNFa* (**f**), measured by qRT-PCR, in isolated enterocytes of *Vil-Cre mir-802*^*fl/fl*^ and control mice (*n* = 5,5 for (**e**) and *n* = 3,3 for (**f**)). Data representative of two independent experiments (**e**) and one experiment (**f**). **g–j** Colonization experiment of 10-week-old pathogen-free *mir-802*^*fl/fl*^ and *Vil-Cre mir-802*^*fl/fl*^ mice that were gavaged with 10^8^ CFU of stationary phase *Salmonella* Typhimurium (strain SL1344) cultures grown overnight. Bacterial levels in spleen, feces and mesenteric lymph nodes (mLNs) were determined by dilution plating of homogenized tissues. Colony formation unit per gram mLN (**g**), colony-forming units per mLN (**h**), colony-forming units per gram of cecal content (**i**), and colony formation unit per gram of spleen (**j**) (*n* = 10,11 for *mir-802*^*fl/fl*^ and *Vil-Cre mir-802*^*fl/fl*^ mice, respectively for (**g**, **h**, **j**) and *n* = 10 per genotype for (**i**), data are from two replicate experiments). Data are plotted as mean ± SD. Significance was evaluated by two-tailed *t*-test (**b**, **e**, **f**), one-way ANOVA with Tukey’s multiple comparisons test (**a**), or Mann–Whitney two-tailed test (**g**–**j**).

To investigate if *mir-802* ablation leads to abnormal phenotypes in the intestine beyond glucose transport, we first explored if morphological differences can be detected in mice lacking *miRr-802*. Crypt and villus sizes and the number of Goblet cells were similar in *Vil-Cre mir-802*^*fl/fl*^ compared to *mir-802*^*fl/fl*^ mice (Supplementary Fig. 2a, b). We next measured the relative *miR-802* levels in different intestinal cell types such as Lgr5+ stem cells, Paneth cells, and enterocytes following FACS sorting. Levels of *miR-802* were highest in Paneth cells (1.7–fold) compared to enterocytes, and much lower in Lgr5+ cells (Fig. 2a). Given the high *miR-802* expression, we next analyzed Paneth cell numbers by immunofluorescence staining using the Paneth cell-specific marker lysozyme (LYZ). Interestingly, we found higher Paneth cell numbers per crypt in the jejunum of *Vil-Cre mir-802*^*fl/fl*^ mice compared to control *mir-802*^*fl/fl*^ animals, while no changes were detected in the proximal ileum (Fig. 2b, Supplementary Fig. 2c). Paneth cells in *Vil-Cre mir-802*^*fl/fl*^ mice were located at the base of intestinal crypts with no indication of Paneth cell metaplasia (Fig. 2b). The increased Paneth cell numbers were also confirmed by FACS analysis of dispersed intestinal epithelial cells (Fig. 2c, Supplementary Fig. 2d), revealing a ≈25% increase in *Vil-Cre mir-802*^*fl/fl*^ compared to *mir-802*^*fl/fl*^ mice. In contrast, enterocyte numbers were similar between groups (54% ± 7 vs. 55% ± 7, respectively). Successful FACS sorting of Paneth cells was validated by qRT-PCR measurements of *Lyz* and *Defa* (Supplementary Fig. 2e). Furthermore, expression analysis of isolated intestinal epithelial cells by RNA-seq and qRT-PCR revealed increased transcript levels of *Lyz*, angiogenin-4 (*Ang4*), phospholipase A2 (*Pla2*), and cryptdins (*Defa 5, 20, 21, 22*), which were all enriched in Paneth cells (Fig. 2d, e). Furthermore, the proteolytic processing enzyme *MMP-7*, which is required for the activation of defensins^18^, was increased in mice with genetic *mir-802* ablation (Fig. 2d). Also, secreted *Reg3γ*, which is also expressed in other gut epithelial cell types and helps to maintain the relative bacterial sterility on the epithelial surface of the jejunum^19^, was also increased (Fig. 2d). This is consistent with a modest increase in *NF-κB* and *Tnfa* expression (Fig. 2f)^20^. Together, these data demonstrate that *miR-802* is broadly expressed in the intestinal epithelium of the jejunum and enriched in intestinal Paneth cells compared to enterocytes, where it regulates Paneth cell expansion.

To evaluate the impact of Paneth cell expansion on murine microbiota, the cecal and ileal microbiota composition of *Vil-Cre mir-802*^*fl/fl*^ and *mir-802*^*fl/fl*^ mice was analyzed by 16S rRNA gene amplicon sequencing. Overall, we measured a similar microbiota composition of the two mouse groups (Supplementary Fig. 2f). A Principal Component and Permutational MANOVA analysis revealed a cage effect and only a minor clustering by phenotype within a cage for the ileum samples (cage effect - *R*^2^: 0.49, p = 0.001; phenotype effect - *R*^2^: 0.03, *p* = 0.56) (Supplementary Fig. 2g). Furthermore, no difference was found in the abundance of individual taxa by testing for differential abundance of zero-radius operational taxonomic units (zOTUs) between the animal groups, indicating that miR-802 has no major effect on the diversity and relative abundance of the microbiome (Supplementary Fig. 2h, i).

Dysregulation of the epithelial tight junction proteins have been linked to altered susceptibility to bacterial enteric pathogens. We measured transcript levels of the tight junction complex, including occludin, claudins and, zonula occludens (*Ocln, Cldn3, Tjp1*), and found slightly increased transcript levels in the small intestine of *Vil-Cre mir-802*^*fl/fl*^ mice, however, on a protein level, only CLDN was modestly upregulated (Supplementary Fig. 2j, k). Also, no functional changes in intestinal permeability were found when measuring fluorescein isothiocyanate-conjugated (FITC) dextran in serum following an oral gavage (Supplementary Fig. 2l). However, protection against enteric salmonellosis has also been associated with increased intestinal defensin^21^ and lysozyme^22^ expression, and increased susceptibility has been reported in models with impaired Paneth cell function^18,23^ or after induction of Paneth cell degranulation^24^. To investigate if this association also holds in mice lacking *mir-802*, we orally administered high doses of virulent *Salmonella* Typhimurium to *Vil-Cre mir-802*^*fl/fl*^ and control *mir-802*^*fl/fl*^ mice. We measured significantly decreased recovery of viable *S*. Typhimurium from the mesenteric lymph nodes (mLN), with similar distribution in the spleen and cecal contents, indicating reduced bacterial dissemination and spread beyond the gut epithelium in the absence of intestinal *miR-802* (Fig. 2g–j). Together, these findings indicate that mice with genetic ablation of *mir-802* in the intestine may be more resistant to virulent *S*. Typhimurium than control mice, thereby suggesting a role for miR-802 in epithelial-derived defensins in the mammalian host defense.

### *miR-802* regulates proliferation and intestinal epithelial cell turnover

**Fig. 3:**
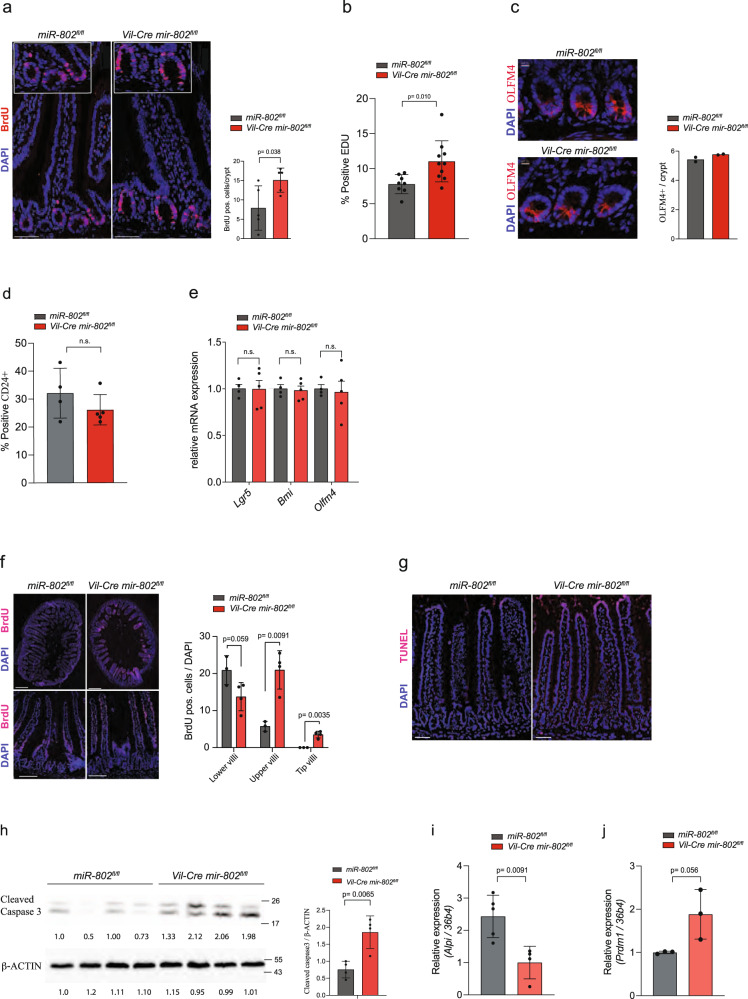
Increased intestinal proliferation in mice with intestine-specific ablation of *mir-802*. **a** Left: Representative stainings of *Vil-Cre mir-802*^*fl/fl*^ and *mir-802*^*fl/fl*^ control mice injected with BrdU 90 min before sacrifice. Sections were stained with BrdU antibodies and DAPI. Right: BrdU positive cells quantified with ImageJ and normalized per crypt number. On average 60 crypts were analyzed per sample. Scale bar: 50 µm, (*n* = 5 per genotype, one experiment). **b** Percent positive EDU cells of isolated jejunum crypts by FACS from *Vil-Cre mir-802*^*fl/fl*^ and *mir-802*^*fl/fl*^ that were injected with 20 mg/kg body weight EDU (*n* = 10,8 *Vil-Cre mir-802*^*fl/fl*^ and *mir-802*^*fl/fl*^ respectively, (one experiment)). **c** Left: Representative stainings of OLFM4 in jejunal intestinal crypts from *Vil-Cre mir-802*^*fl/fl*^ and control mice. Right: Quantification of Olfm4+ cells per crypt. Scale bar: 10 µm, (*n* = 2 per genotype). **d** Quantification of FACS sorted % positive CD24^+,LOW^ population from isolated epithelial cells from the proximal jejunum of *Vil-Cre mir-802*^*fl/fl*^ and controls (*n* = 4,5 per genotype, (one experiment)). **e** Relative mRNA expression of indicated stem cell markers from FACS sorted cells in (**d**) (*n* = 4,5 per genotype, one experiment). **f** Representative images of BrdU labeled epithelial cells (left) and quantitative analysis of epithelial turnover rate by BrdU pulse assay in upper jejunum of *Vil-Cre mir-802*^*fl/fl*^ and *mir-802*^*fl/fl*^ mice (right). For analysis, villi were divided into three segments (lower, upper, tip). Scale bar: 200 µm top images; 50 µm bottom images (*n* = 3,4 for *mir-802*^*fl/fl*^ and *Vil-Cre mir-802*^*fl/fl*^, one experiment. **g** Representative images of two independent experiments of jejunum sections from *Vil-Cre mir-802*^*fl/fl*^ and *mir-802*^*fl/fl*^ mice stained with a Tunel assay. Scale bar: 50 µm. **h** Immunoblot of cell lysates from jejunum of *Vil-Cre mir-802*^*fl/fl*^ and *mir-802*^*fl/fl*^ mice blotted for Cleaved Caspase 3. β-ACTIN was used as a loading control. The quantification of densitometric analysis is shown on the right. Each lane represents a lysate from a different mouse (*n* = 4 per genotype, one experiment). **i**, **j** Relative expression, measured by qRT-PCR, of *alkaline phosphatase* (*Alpi)* (**i**) and *Prdm1* (**j**) in proximal jejunum (total tissue) of *Vil-Cre mir-802*^*fl/fl*^ and *mir-802* ^*fl/fl*^ mice (*n* = 5,4 for *Alpi*, *n* = 3 per group for *Prdm1*, in *mir-802*^*fl/fl*^ and *Vil-Cre mir-802*^*fl/fl*^, respectively, one experiment)). Data are plotted as mean ± SD. Significance was evaluated by unpaired two-tailed *t*-tests (**a**, **b**, **d**, **e**, **h**–**j**), two-tailed *t*-test with Holm-Sidak correction for multiple comparisons (**f**).

Apart from their role in innate immunity and host-defense, Paneth cells also secrete factors such as WNT, NOTCH, EGF, BMP, that create the right microenvironment to sustain and modulate the stem cell niche in homeostatic and stress conditions^16^. To explore whether *miR-802* contributes to the regulation of proliferation, we analyzed the intestines of mice injected with thymidine analog 5′-bromo-2′- deoxyuridine (BrdU). Proliferation rates were increased in jejunal, but not ileal crypts, of adult and newborn mice lacking *mir-802* (Fig. 3a) (Supplementary Fig. 3a, b). In addition, we performed a flow cytometric analysis in crypts after EDU injection in *Vil-Cre mir-802*^*fl/fl*^ mice and confirmed the increased numbers of proliferating cells (Fig. 3b). Interestingly, the overall length of the intestine was longer in *Vil-Cre mir-802*^*fl/fl*^ mice compared to control *mir-802*^*fl/fl*^ (Supplementary Fig. 3c–e). To investigate whether *miR-802* directly affects intestinal stem cell (ISC) numbers we quantitatively assessed OLFM4-positive cells per crypt in jejunal sections. This analysis showed no alterations in ISC numbers in *Vil-Cre mir-802*^*fl/fl*^ compared to control mice (Fig. 3c). We also FACS sorted ISCs directly from *Vil-Cre mir-802*^*fl/fl*^ and controls and obtained similar enriched ISC fractions in both groups (Fig. 3d, Supplementary Fig. 3f). Furthermore, gene expression analysis of stem cell markers (*Lgr5*, *Bmi*, and *Oflm4*) from sorted cells were similar in *Vil-Cre mir-802*^*fl/fl*^ and control mice, thereby confirming the OLFM4 cell quantification (Fig. 3e).

The epithelium of the small intestine represents the most rapidly self-renewing tissue in adult mammals, with homeostatic self-renewal controlled by an equilibrium between the proliferation of progenitor cells in the crypts and apoptosis at the tips of the villi^25,26^. We hypothesized that the increased proliferation, yet normal crypt and villus length, could be reconciled by a balance of cell proliferation and death. This model would predict an increased migration of epithelial cells from the crypts to the villi countered by increased shedding at the villi tips. To test this hypothesis, we performed BrdU-pulse labeling experiments by injecting *Vil-Cre mir-802*^*fl/fl*^ and *mir-802*^*fl/fl*^ mice with BrdU 36 h before tissue fixation and anti-BrdU staining. This experiment revealed enhanced epithelial cell turnover in the jejunum, as shown by a faster migration of labeled cells towards the tips of the villi in *Vil-Cre mir-802*^*fl/fl*^ mice compared to control mice (Fig. 3f). This finding was further supported by increased Tunel staining at the tips of the villi in *Vil-Cre mir-802*^*fl/fl*^ mice (Fig. 3g), furthermore, Cleaved Caspase 3 immunoblotting also demonstrated increased apoptosis in the jejunum of *Vil-Cre mir-802*^*fl/fl*^ mice (Fig. 3h). Together, these findings raised the question of whether the increased turnover of enterocytes could lead to an impairment in their differentiation, as suggested by the reduced expression of glucose transporters, which is a hallmark of dedifferentiation in pancreatic β-cells^27^. Expression analysis of the enterocyte-specific marker alkaline intestinal phosphatase (*Alpi*)^28,29^, expressed in differentiated enterocytes but not in immature crypts, showed a significant reduction in *Vil-Cre mir-802*^*fl/fl*^ jejunal tissue compared to control jejunum (Fig. 3i). Furthermore, we measured increased levels of *Prdm1*, a transcriptional repressor that delays postnatal epithelial maturation^30,31^ (Fig. 3j). These data imply that *miR-802* in the intestinal epithelium increases proliferation, accelerates epithelial cell turnover, perturbs absorptive enterocyte differentiation, and increases apoptosis.

### Gene expression and *miR-802* target identification in the intestinal epithelium

**Fig. 4:**
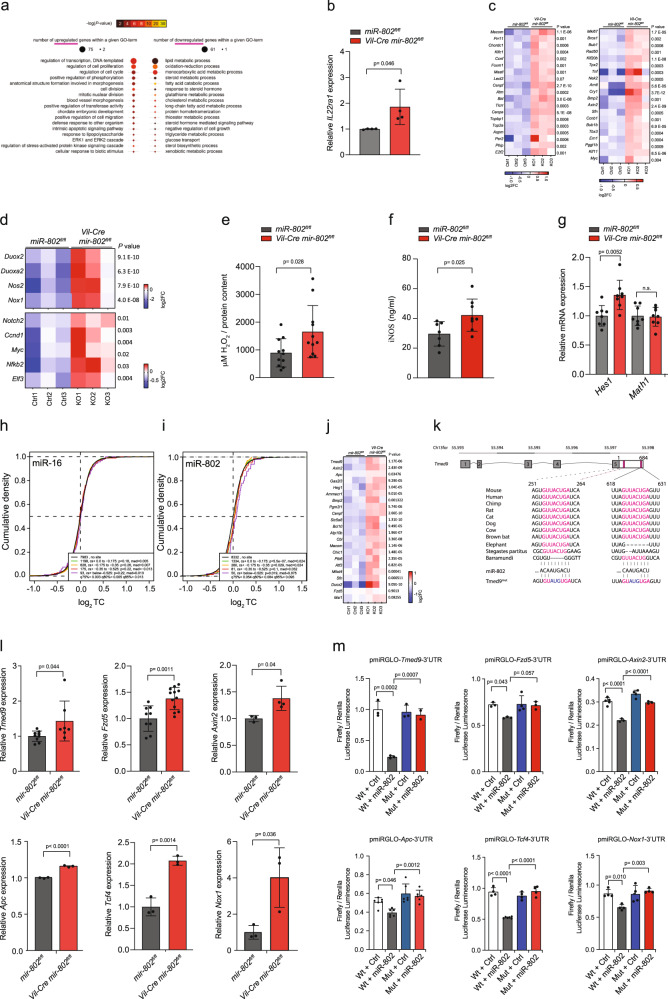
Transcriptome analysis of isolated intestinal epithelial cells. **a** GO plots from RNAseq data representing the upregulated (left) and downregulated biological processes (right). **b** Relative expression of *IL22ra1*, measured by qRT-PCR, in isolated crypts from upper jejunum (*n* = 4 per group, one experiment). **c** Heatmap of transcript expression from RNAseq data of positive regulators of the cell cycle (GO 0051726) shown as Log2FC (*n* = 3). **d** Heatmap of transcript expression from RNAseq data of ROS-producing genes (top) and Notch2 and downstream targets (bottom) shown as Log2FC (*n* = 3). **e**, **f** ROS (H_2_O_2_) (**e**) and iNOS (**f**) production in the upper jejunum of *Vil-Cre mir-802*^*fl/fl*^ and *mir-802*^*fl/fl*^ mice (*n* = 11 per genotype for ROS, two independent experiments) (*n* = 8, 7 per genotype for iNOS, respectively, one experiment). **g** Relative expression measured by qRT-PCR of Notch targets *Hes1* and *Math1* in isolated enterocytes of upper jejunum (*n* = 8 per genotype, one experiment). **h**, **i** Global *miR-802* target gene regulation from RNAseq (*n* = 3 per group). Cumulative density blots of RNA-seq data for miR-802 (**h**) and miR-16 targets (**i**) (negative control), grouped by context score (cs+ based on Target Scan 7.1). **j** Heatmap from RNAseq data of 22 most differentially expressed target genes of *miR-802* shown as Log2FC (*n* = 3). **k** Gene organization and evolutionary conservation of *miR-802* target sites in *3*′*UTR* of *Tmed9*. **l** Validation of indicated *miR-802* target gene expression by qRT-PCR in isolated crypts of upper jejunum from *Vil-Cre mir-802*^*fl/fl*^ and *mir-802*^*fl/fl*^ mice. *n* = 9,9,3,3,3,3 for WT crypts, 7,12,4,3,3,3 for KO crypts, respectively, one experiment. **m** Dual-luciferase assays for *miR-802* targets (*Tmed9*, *Fzd5*, *Axin2*, *Apc*, *Tcf4*, *Nox1*) in HEK293T transfected with plasmid-pmirGLO-WT 3′UTR (Wt) or plasmid-pmirGLO-mutant 3′-UTR, and miRNA mimics for *miR-802* or control (Ctrl). Firefly luciferase activity was normalized to Renilla luciferase activity and expressed relative to control. *n* = 3,3,5,6,4,4 for Wt + Ctrl, 3,3,3,6,6,4 for Wt + miR-802, 3,4,4,6,4,4 for Mut + Ctrl and 2,3,3,6,4,4 for Mut + miR-802, one experiment. Data are plotted as mean ± SD. Significance was evaluated by a two-tailed *t*-test (**b**, **e**–**g**, **l**), or one-way ANOVA followed by Tukey’s multiple comparison test (**m**).

To address the molecular basis for the phenotypic alterations observed in *mir-802* null mice, we performed RNA sequencing (RNA-Seq) analysis on isolated jejunal epithelial cells of *Vil-Cre mir-802*^*fl/fl*^ and *mir-802*^*fl/fl*^ control animals. We determined that 242 transcripts were downregulated with a cutoff of 0.5 log2 ratio (*p*-value 0.01) in *Vil-Cre mir-802*^*fl/fl*^ cells compared to controls, while 149 genes were upregulated using the same cutoff (Supplementary Fig. 4a). Gene Ontology (GO) enrichment analysis of differentially expressed genes (DEGs) identified proliferation and cell cycle as top upregulated biological process, which included genes of the Wnt–β-catenin pathway (i.e., *Ccnd1, Myc*), IL22ra1 signaling, known to stimulate Wnt expression and intestinal proliferation (Fig. 4a–c)^32^. In addition, we found strictly proliferation-dependent transcripts that are known positive regulators of cell proliferation (e.g., *Mecom, Foxm1, Atm, E2f2, MKi67, Bmp2, Tbx3, Axin2*) (Fig. 4c)^33–41^. We also identified increased levels of ROS-producing enzymes in *Vil-Cre mir-802*^*fl/fl*^ mice, including *Nox1*, which harbors a 7mer-A1 *miR-802* binding site in its 3′ UTR (Fig. 4d). Consequently, we measured increased levels of ROS and iNOS in the intestines of mice with *mir-802* ablation (Fig. 4e, f). Since ROS-producing oxidases have been shown to act as modulators of Notch signaling in the intestine^42^, we measured *Notch* expression in jejunal crypts and found them to be increased. Consistent with *Notch* upregulation we measured a modest increase of the target *Hes1*, however, *Math1* was not repressed, suggesting that not all downstream components of the Notch pathway were activated in *Vil-Cre mir-802*^*fl/fl*^ mice (Fig. 4g). Interestingly, RNA seq analysis also showed upregulation of several positive regulators of Paneth cell differentiation (*Apc, Prdm1, Lgr4, Nox1, Il22ra1*), maturation (*Ppard*), and crypt localization (*EPHB2/3*) in the intestine of *Vil-Cre mir-802*^*fl/fl*^. Lastly, we identified a total of 21 antioxidant genes with decreased expression levels in *Vil-Cre mir-802*^*fl/fl*^ mice compared to controls (Supplementary Fig. 4b). They likely contribute to the increase in intestinal ROS, although none of these transcripts harbor *miR-802* binding sites in their 3′UTR. Together, these data suggest that loss of *miR-802* activates several pathways regulating epithelial homeostasis in the intestine, including Wnt and Notch signaling and components of the ROS generating system.

We also identified many downregulated biological processes, amongst them metabolic pathways, in agreement with a total of 46 nutrient transporters that exhibited reduced expression in mice lacking *mir-802* in the intestine (Supplementary Fig. 4c). This extensive downregulation of transporters further supports our finding that the intestinal epithelium of *mir-802KO* mice is not fully differentiated.

Direct target transcripts of *miR-802* are expected to be derepressed and therefore upregulated in cells with genetic *mir-802* ablation. Transcripts that carry *miR-802* motifs showed significant upregulation in *Vil-Cre mir-802*^*fl/fl*^ mice compared with transcripts that do not (*P* < 10^–6^, two-sided Mann–Whitney test), while no seed enrichment was observed for predicted targets of the ubiquitously expressed *miR-16* (Fig. 4h, i). RNA-Seq analysis identified only 22 potential direct target transcripts of *miR-802* that were regulated in intestinal cells of *Vil-Cre mir-802*^*fl/fl*^ mice (Fig. 4j) Interestingly, we did not measure a significant upregulation of predicted *miR-802* targets, such as *Hnf1b, Gata4*, and *Msl1*, which are known to affect Paneth cell and enterocyte differentiation^43–45^. Lastly, *A1atr*, a previously reported target gene, was not derepressed in the liver and colon^8,46^.

To further characterize the extent to which *miR-802* is capable of repressing the endogenous transcripts of the top predicted targets, we overexpressed *miR-802* at different MOIs and measured endogenous target transcript levels^7^. Surprisingly, in contrast to a previous report^8^, *Hnf1b* showed little repression, whereas the transmembrane emp24 domain-containing protein-9 (*Tmed9*) transcript was by far the most sensitive to dose-dependent inhibition by *miR-802* (Supplementary Fig. 4d). *Tmed9* harbors two evolutionary-conserved 8-mer seeds (Fig. 4k), is the top predicted target transcript of *miR-802* in TargetScan^7^, and was also identified as the top upregulated target transcript in our RNA-seq data comparing intestinal epithelial cells from *Vil-Cre mir-802*^*fl/fl*^ and control mice (Fig. 4j). *Tmed9* is a member of the EMP24/GP25L/Erp (endomembrane protein precursor of 24 kD) family, which are abundant proteins of the early secretory pathway and that are involved in the transportation and delivery of diverse membrane and secretory proteins. Importantly, Tmed proteins have been shown to promote Wnt secretion and β-catenin signaling^47–49^. Since Wnt signaling plays a key role in the regulation of intestinal stem cells and homeostatic control of the gut epithelium^15,16^ we investigated if other predicted *miR-802* targets could influence the Wnt signaling network and therefore might explain the phenotype of *Vil-Cre mir-802*^*fl/fl*^ mice. Indeed, we identified and independently validated the derepression of six conserved targets in isolated crypts of *Vil-Cre mir-802*^*fl/fl*^ compared to *mir-802*^*fl/fl*^ mice. They included *Tmed9*, the *Wnt* receptor *Fzd5*, two members of the β-catenin destruction complex (*Axin2* and *Apc*), *Tcf4* a downstream target of Wnt signaling, and the NADPH oxidase1 (*Nox1*) (Fig. 4l). To test whether the predicted miR-802 target sites in the 3′ UTR of *Tmed9*, *Fzd5*, *Axin2*, *Apc*, *Tcf4*, and *Nox1* mRNA were responsible for their derepression in the absence of *miR-802*, we cloned their putative *3*′ *UTRs* downstream of a luciferase reporter gene and co-transfected this vector into HEK293 cells, which do not express *miR-802* and are sensitive to changes in reporter activity, along with a vector driving the expression of *miR-802* or a control vector (Fig. 4m, Supplementary Fig. 4e). Luciferase activity of cells with forced *miR-802* overexpression was decreased by ≈80%, 20%, 30%, 20%, 50%, and 30%, respectively, relative to that of cells that were co-transfected with control vectors. Point mutations in the seed region of the *miR-802* target site (mut) abolished the repression of *miR-802* on luciferase activity (Fig. 4m). These data show that *Tmed9*, *Fzd5*, *Axin2*, *Apc*, *Tcf4*, and *Nox1* are direct targets of *miR-802* in epithelial cells and that their repression is mediated by *miR-802* target sites in their respective *3*′*UTRs*. In conclusion, we identified and validated six *miR-802* targets related to Wnt signaling with a possible role in the regulation of intestinal stem cell proliferation.

### *Tmed9* is a *miR-802* target that modulates Wnt/β-catenin signaling

**Fig. 5:**
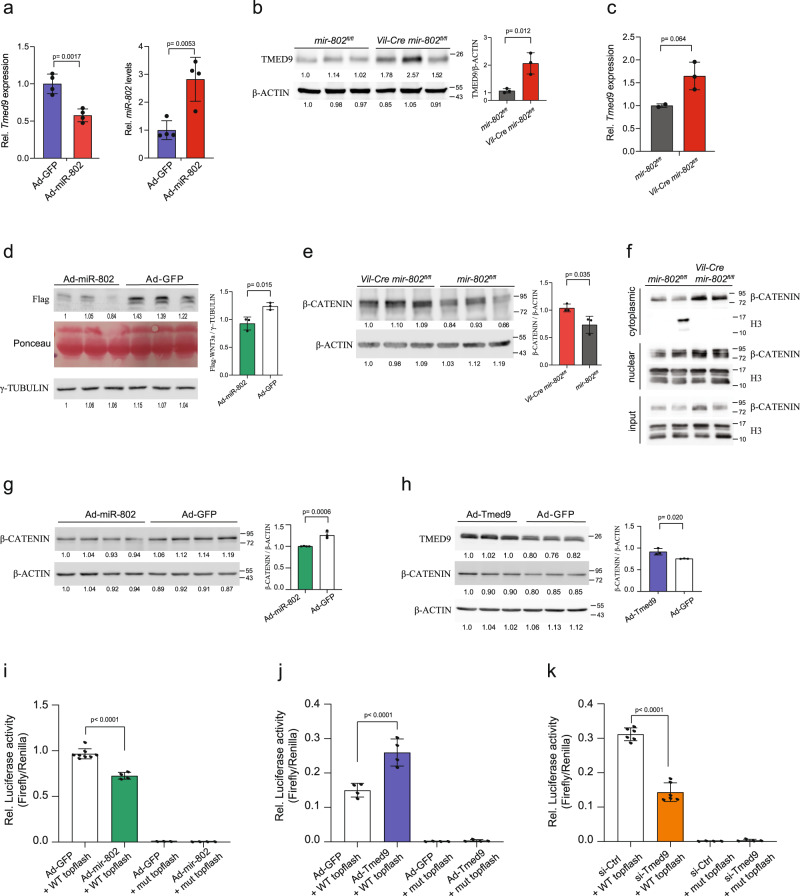
*TMED9* is a *miR-802* target that modulates Wnt/β-catenin signaling. **a** qRT-PCR of *Tmed9* (right) and *miR-802* (left) of mouse small intestine-derived organoids infected with miR-802 adenovirus compared to control adenovirus (*n* = 4 per group, two independent experiments). **b** Immunoblot of TMED9 protein in lysates of upper jejunum from *Vil-Cre mir-802*^*fl/fl*^ and *mir-802*^*fl/fl*^ control mice. β-ACTIN was used as a loading control. Quantitative analysis of densitometric measurements is shown on the right. Each lane represents a lysate from a different mouse (*n* = 3 per genotype). Representative data of two independent experiments. **c** *Tmed9* transcript levels, measured by qRT-PCR, in FACS-sorted Paneth cells of *Vil-Cre mir-802*^*fl/fl*^ *mice* compared to *mir-802*^*fl/fl*^ controls (one independent experiment, each dot represents 3 pooled mice, (*n* = 3,2 per genotype)). **d** Immunoblot analysis of supernatant from C2BBe1 cells expressing FLAG-tagged WNT3a that were infected with either Ad-miR-802 or control Ad-GFP. The image of the Ponceau stained gel is shown as a loading control. γTUBULIN of cell lysates was used as an additional loading control. Quantitative analysis of densitometric measurements is shown on the right (*n* = 3 per group, two independent experiments). **e** Immunoblot analysis of β-CATENIN from isolated epithelial cells of upper jejunum of *Vil-Cre mir-802*^*fl/fl*^ and *mir-802*^*fl/fl*^ mice. Quantitative analysis of densitometric measurements is shown on the right. Each lane represents a lysate from a different mouse (*n* = 3 per genotype, one experiment). **f** Cell fractionation followed by immunoblotting of β-CATENIN from isolated crypts. H3 was used as a nuclear marker. Each lane represents a lysate from a different mouse (*n* = 2 per genotype, two independent experiments). **g**, **h** Immunoblot analysis of β-CATENIN in C2BBe1 cells infected with Ad-miR-802 (**g**) or Ad-Tmed9 (**h**) and control Ad-GFP adenoviruses. β-ACTIN was used as a loading control. Quantitative analysis of densitometric measurements is shown on the right (*n* = 3 per group, one experiment). **i**, **j** C2BBe1 cells were transduced with Ad-miR-802 (**i**) or Ad-Tmed9 (**j**) or control Ad-GFP virus, and transfected after with TOPFlash or FOPFlash plasmids together with Renilla luciferase (internal control). After 36 h cells were lysed and assayed using the dual-luciferase reporter system (*n* = 8,4,3,4 for (**i**) and *n* = 4 for (**j**) (one experiment)). **k** C2BBe1 cells were transfected with siTmed9 or control siRNAs and TOPflash or FOPflash plasmids together with Renilla luciferase (internal control) (*n* = 6,6,4,4, one experiment). Data are plotted as mean ± SD. Statistical significance was evaluated by unpaired two-tailed *t*-test (**a**–**e**, **g**, **h**) or by one-way ANOVA followed by Tukey’s multiple comparison test (**i**–**k**).

To further establish that *Tmed9* is a *miR-802* target in the intestine we generated mouse small intestine-derived organoids and overexpressed *miR-802* using recombinant adenovirus. Results showed that *Tmed9* is repressed upon *miR-802* overexpression (Fig. 5a). In addition, TMED9 protein levels, determined by Western blotting, were derepressed in intestines of *Vil-Cre mir-802*^*fl/fl*^ and in jejunal *Vil-Cre mir-802*^*fl/fl*^ derived organoids compared to respective wild-type controls (Fig. 5b, Supplementary Fig. 5a). Moreover, *Tmed9* transcript levels, measured by qRT-PCR, were increased in FACS-sorted Paneth cells of *Vil-Cre mir-802*^*fl/fl*^ mice compared to *mir-802*^*fl/fl*^ controls (Fig. 5c). These results confirm *Tmed9* as a *miR-802* target in the small intestine and Paneth cells.

Paneth cells are the major epithelial WNT producers within the intestinal crypts and a previous study has shown TMED9 to affect WNT secretion^48,50–52^. We, therefore, hypothesized that *miR-802* may modulate WNT secretion and signaling through the action of TMED9. Since WNT3A is post-translationally modified by palmitoylation^53^, which renders the protein hydrophobic and unstable in aqueous solutions^54^, we infected human intestinal epithelial cells (C2BBe1) expressing a recombinant Flag-tagged WNT3A with recombinant adenovirus Ad-miR-802 or control virus (Ad-GFP). After 36 h the medium was collected and the flagged WNT protein levels were analyzed by immunoblotting. The analysis revealed a robust 40% decrease in WNT secretion from cells that were transduced with Ad-miR-802 compared to controls (Ad-GFP) (Fig. 5d). We next analyzed the downstream effectors of Wnt signaling by immunoblotting β-CATENIN in the jejunum of *Vil-Cre mir-802*^*fl/fl*^ and control mice. Levels of β-CATENIN in *Vil-Cre mir-802*^*fl/fl*^ mice were increased by ≈25% compared to WT mice (Fig. 5e). Besides, we found increased nuclear localization of β-CATENIN in isolated crypts (Fig. 5f). Furthermore, C2BBe1 cells infected with Ad-miR-802 had decreased β-CATENIN expression compared to control Ad-GFP-infected cells (Fig. 5g). TMED9 overexpression also resulted in a modest increase in β-CATENIN expression (Fig. 5h). To further explore whether *miR-802* and *Tmed9* affect downstream β-catenin-dependent transcription, we infected C2BBe1 cells with Ad-miR-802 or control adenovirus (Ad-GFP), followed by transfection of a luciferase construct containing four native TCF/LEF binding sites (TOPflash) or its negative-control counterpart with mutated LEF/TCF binding sites (FOPflash)^54,55^. TCF/LEF transcriptional activity was decreased in cells in which miR-802 was overexpressed (Fig. 5i). Furthermore, adenoviral overexpression of Tmed9 resulted in enhanced TCF/LEF activity compared to controls (Ad-GFP), whereas silencing of *Tmed9* by RNAi decreased reporter activity compared to controls (Fig. 5j, k). Luciferase activity could only be measured in TOPflash- but not in FOPflash-transfected cultures (Fig. 5i–k). Taken together, these results indicate that *miR-802* and its target *Tmed9* are, respectively, negative and positive regulators of WNT-β-CATENIN secretion and signaling.

### *miR-802* site mutations in *Tmed9* partially phenocopy *mir-802* ablation

**Fig. 6:**
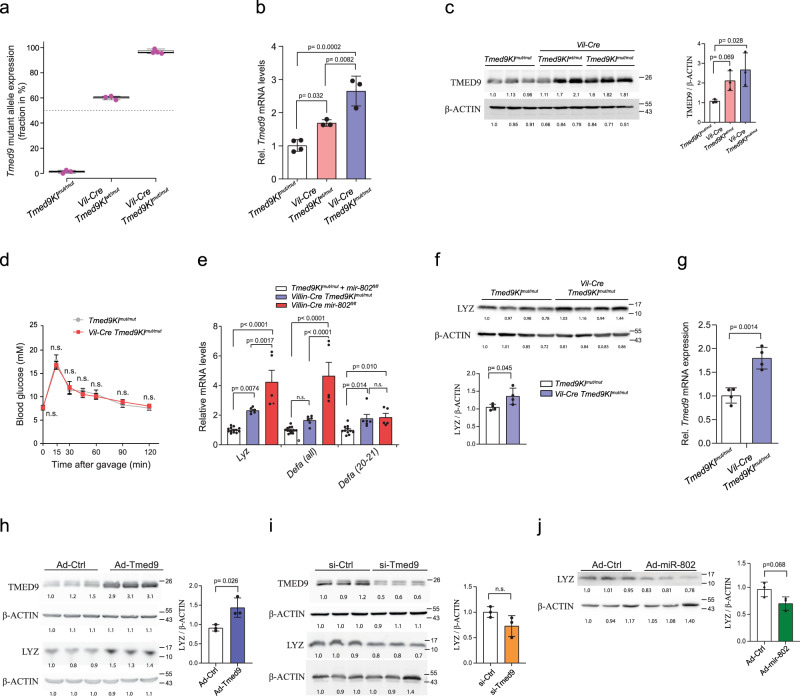
Mutation of *miR-802* binding sites in*Tmed9* is sufficient to phenocopy the *Vil-Cre mir-802*^*fl/fl*^ Paneth cell phenotype. **a** Fraction (%) of *Tmed9* mutant allele expression on *Tmed9* expression in the intestinal epithelium of *Vil-Cre Tmed9KI*^*mut/mut*^, *Vil-Cre Tmed9KI*^*wt/mut*^, and control *Tmed9KI*^*mut/mut*^ mice. Box plot defined by minima (0, 58.43, 95.46), median (1.377, 60.55, 96.21), maxima (2.5, 61.39, 98.98), 25% percentile (0,58.43, 95.6), 75% percentile (2.5, 61.39, 98.33), range (2.5, 2.957, 3.520), mean (1.292, 60.12, 96.71) for genotypes *Tmed9KI*^*mut/mut*^*,Vil-Cre Tmed9KI*^*wt/mut*^, *Vil-Cre Tmed9KI*^*mut/mut*^ respectively with line shown at mean (*n* = 3, 3, 4 respectively per genotype). **b** Relative mRNA expression, measured by qRT-PCR, of *Tmed9* in the isolated intestinal epithelium of *Vil-Cre Tmed9KI*^*mut/mut*^, *Vil-Cre Tmed9KI*^*wt/mut*^, and control *Tmed9*^*mut/mut*^ mice (*n* = 3,3,4 for each genotype respectively). Data are representative of three independent experiments. **c** Immunoblot analysis of TMED9 in the jejunum of *Vil-Cre Tmed9KI*^*mut/mut*^ *Vil-Cre Tmed9KI*^*wt/mut*^, and control *Tmed9KI*^*mut/mut*^ mice. β-ACTIN was used as a loading control. Quantitative densitometric analysis is shown on the right. Each lane represents a lysate from a different mouse (*n* = 3 per genotype). Data are representative of two independent experiments. **d** Oral glucose tolerance test of *Vil-Cre Tmed9KI*^*mut/mut*^ and control *Tmed9KI*^*mut/mut*^ mice (*n* = 7 for each group, two independent experiments). **e** Relative mRNA expression levels, measured by qRT-PCR, of antimicrobial peptides in jejunum of *Vil-Cre Tmed9KI*^*mut/mut*^ and *Vil-Cre mir-802*^*fl/fl*^ compared to control mice (*Tmed9KI*^*mut/mut*^ and *mir-802*^*fl/fl*^) (*n* = 13, 6, 5 for *Tmed9KI*^*mut/mut*^ and *mir-802* ^*fl/fl*^, *Vil-Cre Tmed9KI*^*mut/mut*^, and for *Vil-Cre mir-802* ^*fl/fl*^, respectively for each group). Data are representative of two independent experiments. **f** Immunoblot analysis of LYZ in isolated enterocytes from upper jejunum of *Vil-Cre Tmed9KI*^*mut/mut*^ and *Tmed9KI*^*mut/mut*^ littermates. The quantification of densitometric analysis is shown on the right. Each lane represents a lysate from a different mouse (*n* = 4 per genotype, two independent experiments). **g** *Tmed9* transcript levels, measured by qRT-PCR, in FACS-sorted Paneth cells of *Vil-Cre Tmed9KI*^*mut/mut*^ *mice* compared to *Tmed9KI*^*mut/mut*^ controls (*n* = 4 per genotype, one experiment). **h**, **i** Immunoblot analysis of LYZ in C2BBe1 cells that were infected with Ad-Tmed9 (**h**) or transfected with siRNAs targeting Tmed9 or control siRNAs (**i**). β-ACTIN was used as a loading control. Quantitative densitometric analysis is shown on the right (*n* = 3 per group, one experiment). **j** Immunoblot analysis of LYZ in C2BBe1 cells infected with Ad-miR-802 and control (Ad-GFP) adenovirus. β-ACTIN was used as a loading control. Quantitative densitometric analysis is shown on the right (*n* = 3 per group). Data are representative of two independent experiments. Data are plotted as mean ± SD. Statistical significance was evaluated by descriptive statistics (**a**) unpaired two-tailed *t*-tests (**f**–**j**), by one-way ANOVA followed by Tukey’s multiple comparison test (**b**, **c**, **e**), or two-way ANOVA for repeated measures with Sidak’s multiple comparisons test (**d**).

Most miRNAs function by repressing the expression of hundreds of targets, whose additive effect can impart strong phenotypic consequences^1,3^. So far, there are only a few examples known in which a specific deregulated target can explain the phenotypic effects observed in genetic models with loss of miRNA function^56,57^. Since *Tmed9* was the most regulated *miR-802* target in vivo and the most sensitive to *miR-802* repression in vitro, we explored the contribution of *Tmed9* deregulation vs. that of hundreds of other mRNAs with conserved *miR-802* sites by generating a conditional knock-in mouse model in which both *miR-802* sites in the endogenous *3*′*UTR* of *Tmed9* were mutated (*Tmed9KI*^*mut/mut*^) to disengage *Tmed9* from *miR-802* repression (Fig. 4k and Supplementary Fig. 6a). To study the contribution of *Tmed9* to the *Vil-Cre mir-802*^*fl/fl*^ phenotype, we crossed (*Tmed9KI*^*mut/mut*^) with *Vil-Cre* transgenic mice to generate heterozygous or homozygous animals (*Vil-Cre Tmed9KI*^*mut/wt*^ *or Vil-Cre Tmed9KI*^*mut/mut*^, respectively). RNA sequencing and qRT-PCR of the intestinal epithelium was analyzed to determine the expression of wild-type (*WT*) and mutant *Tmed9* alleles from *Vil-Cre Tmed9KI*^*mut/wt*^ *or Vil-Cre Tmed9KI*^*mut/mut*^ mice, demonstrating gene dosage-dependent expression of the mutant allele compared to the WT allele in heterozygous and homozygous mutant animals (Fig. 6a, b). TMED9 protein levels were also evaluated, revealing a smaller increase (≈40%) (Fig. 6c). Increased *Tmed9* levels did not affect *miR-802* expression or expression levels of other Tmed family members (Supplementary Fig. 6b, c). Furthermore, *Tmed9* derepression was restricted to the intestine, thereby validating our conditional, intestine-specific *Vil-Cre Tmed9KI*^*mut/mut*^ mouse model (Supplementary Fig. 6d).

**Fig. 7:**
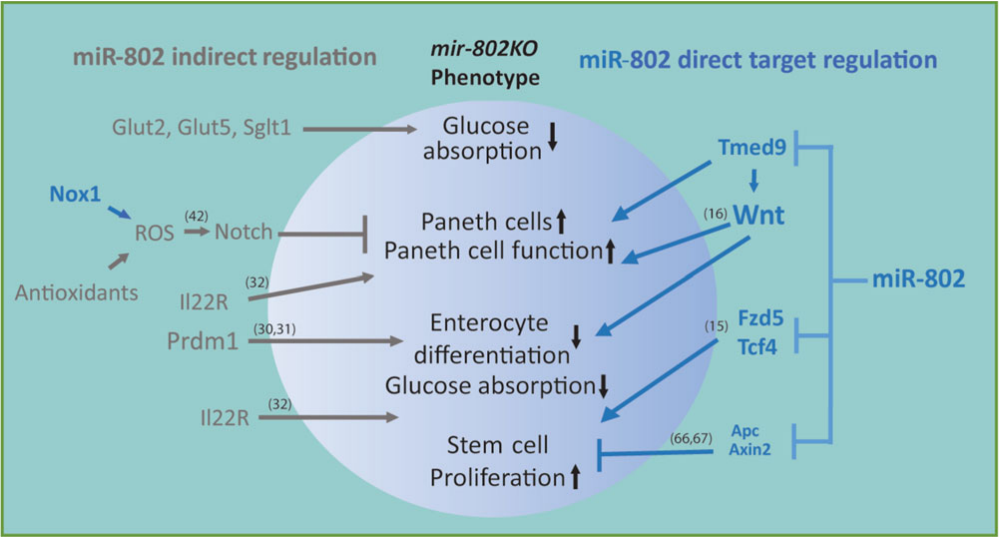
Schematic representation of a model depicting direct and indirect mechanisms of *miR-802* action in intestinal epithelium homeostasis. Blue arrows indicate findings made in this study, previously reported regulatory pathways are shown in gray and are referenced.

We next investigated whether *Tmed9* derepression in the intestinal epithelium of *Vil-Cre Tmed9KI*^*mut/mut*^ *mice* would partially phenocopy *Vil-Cre mir-802*^*fl/fl*^ animals. No metabolic abnormalities were observed in response to an oral glucose challenge (Fig. 6d). Furthermore, RNA seq analysis of intestinal epithelium from *Vil-Cre Tmed9KI*^*mut/mut*^ and control *Tmed9KI*^*mut/mut*^ mice did not show changes in the expression of solute carrier transporters or *Alpi* (Supplementary Fig. 6e), indicating that enterocyte differentiation was not affected by *Tmed9* overexpression. Nonetheless, expression of *defensins* and *Lyz* was increased in *Vil-Cre Tmed9KI*^*mut/mut*^ animals (Fig. 6e, f), in line with increased *Tmed9* levels in sorted Paneth cells (Fig. 6g). To obtain further evidence that TMED9 regulates the secretion of antimicrobial peptides, we perturbed TMED9 levels in C2BBe1 cells using adenoviral overexpression or RNAi and compared LYZ concentrations in the supernatant. Overexpression of TMED9 increased LYZ secretion in the supernatant, whereas silencing of TMED9 resulted in borderline decreased LYZ expression (Fig. 6h, i). In addition, overexpression of *miR-802* reduced LYZ secretion (Fig. 6j). However, *Vil-Cre Tmed9KI*^*mut/mut*^ mice were not protected from *Salmonella* invasion compared to *Tmed9KI*^*mut/mut*^ mice (Supplementary Fig. 6f), most likely due to a smaller increase in defensin expression compared to *Vil-Cre mir-802*^*fl/fl*^ mice (Fig. 6e). Similarly, Paneth cell numbers were similar (Supplementary Fig. 6g) and epithelial proliferation and apoptosis rates, as measured by in vivo BrdU labeling, Cleaved Caspase-3 immunoblotting and gene expression, respectively, were unchanged in *Tmed9KI*^*mut/mut*^ mice (Supplementary Fig. 6h–j), consistent with a normal differentiated epithelium in *Vil-Cre Tmed9KI*^*mut/mut*^ mice. Lastly, we did not observe upregulation of oxidative or downregulation of antioxidant transcripts in the gut epithelium of *Tmed9KI*^*mut/mut*^ mice (Supplementary Fig. 6k, l). Together, these data provide in vivo evidence for a specific role for the *miR-802 – Tmed9* axis in the regulation of Paneth cell function (Fig. 7).

## Discussion

Our study has demonstrated that *miR-802* plays an important role in epithelial intestinal homeostasis, which is supported by high expression levels of *miR-802* in Paneth cells and enterocytes as well as altered phenotypes in these cell types. Interestingly, during evolution *mir-802* first emerged in bony fish during the Silurian period 430 million years ago, when the short elasmobranch digestive system expanded into an intestine, which is absent in non-teleost fish^58,59^. Many bony fish also contain Paneth cells and enteroendocrine cells in their crypts. Furthermore, at least one of the *Tmed9* target sites in the *3*′*UTR* is present in bony fish, whereas the *miR-802* sites are absent in Wnt signaling genes *Fzd5*, *Axin2*, *Apc*, and *Tcf4* (Fig. 4k, Supplementary Fig. 4j).

Our study in *miR-802*-deficient mice uncovered a pleiotropic phenotype manifested by increased proliferation, increased Paneth cell numbers/function, and partly dedifferentiated enterocytes (Fig. 6k). There is increasing evidence that an intricate functional relationship exists between Wnt and Notch signaling during the assignment of particular cell fates, for stem cell maintenance, and a proper balance of differentiation between secretory and absorptive cell lineages^15^. Genetic studies in mice have shown that Wnt signals are a major driver of a stem-cell/progenitor gene program and also confer competence for secretory fate decision, most notably by promoting a Paneth-cell maturation program^16,50–52,60^. On the other hand, Notch signaling negatively regulates secretory cell differentiation through repression of *Math1/Atoh1*^61^, whereas in the absence of Notch, stem cells preferentially generate secretory cells at the expense of absorptive cells^62^. Notch activity is required for maintaining the correct balance of Wnt signaling in the crypt, which allows for simultaneous maintenance of ISCs, proliferation, and differentiation^63^. The increased proliferation rate and Paneth cell numbers in the jejunum of mice lacking *mir-802* is likely mediated by increased secretion of Wnt in the intestinal crypts, and by the activation of their downstream effectors LGR5 and TCF4^64,65^. Our finding that *Notch* expression is elevated in the intestines of mice lacking *mir-802* is seemingly contradictory, and the fact that we could measure only small or no responses in the classical Notch targets *Hes1* and *Math1* is indicative that the Notch pathway is not dominant. We also show that *miR-802* is a negative regulator of Wnt signaling, by repressing *Tmed9* expression in Paneth cells, thereby inhibiting Wnt (and defensin) secretion. In addition to Wnt inhibition, *miR-802* also directly represses components of the Wnt signaling cascade, most notably *Fzd5* and downstream target *Tcf4*. However, it is worth noting that *miR-802* also targets *Axin2* and *Apc*, two negative regulators of Wnt signaling, and components of the β-catenin degradation complex^66,67^. In addition, we also observed a marked overexpression of the *Il22ra1* receptor complex that is essential for Wnt secretion, epithelial cell growth, and Paneth cell function, including the production of antimicrobial peptides^32,68^. Together, these findings are in line with the role of miRNAs in buffering pathway activities at different levels by simultaneously dampening the expression of both positive and negative regulators, thereby avoiding runaway pathway activation and preventing stochastic fluctuations in signaling^69^.

In addition to the complex and intricate regulation of major signaling pathways regulating intestinal epithelial homeostasis, *miR-802* also dampens the expression of several ROS-producing genes. Indeed, we noted that ablation of *miR-802* results in increased ROS and iNOS levels. Emerging evidence in the cardiac system and intestine indicates that Notch and Wnt/β-catenin pathways are influenced by Nox-derived ROS^42,70^, suggesting that this pathway may also contribute to the observed *mir-802KO* phenotype.

Our study identified *miR-802* and its target *Tmed9* as a regulator of Paneth cell number and antimicrobial peptide expression. We show that increased TMED9 levels not only promote WNT secretion but also enhance the production of LYZ and defensins from Paneth cells. The strongest evidence for Tmed9’s role in Paneth cell secretion comes from in vivo studies in mutant *Tmed9 KI* mice that exhibit a selective upregulation of *Tmed9* and identified increased *Lyz* and defensin levels in the absence of increased Paneth cell numbers. Paneth cells are responsible for the host defense of the intestinal tract by secreting antimicrobial peptides as a first line of defense. Enhanced Paneth cell function in *mir-802* knockout mice was demonstrated in *S*. Typhimurium infection experiments, showing partial protection in *Vil-Cre mir-802*^*fl/fl*^ mice. These data are consistent with previous studies in which protection against *Salmonella* infection was demonstrated in transgenic mice overexpressing a single human defensin (HD5) or lysozyme^21,22^, or following treatment of recombinant antimicrobial human β-defensins hBD-1 and hBD-2^71^. However, we stress that the *Salmonella* findings merely provide associations and further studies and models are warranted to establish a cause-and-effect mechanistic relationship linking PC changes in *mir-802KO* mice to the resistance to microbial infection. Additional mechanisms may also be responsible for the heightened antimicrobial defense in mice with *mir-802* ablation, including the upregulation of *Il22ra1* and *Reg3γ*, consistent with studies showing that *Il22* activates *Reg3γ* and that this pathway is protective against mucosal *Salmonella* infection in vivo^20,72^. However, the precise role of *miR-802* in the antimicrobial defense warrants further and more detailed investigations in gram-positive and negative invasion models.

Several studies have demonstrated the ability of intestinal commensals to induce mucosal innate immune effectors, including *Reg3γ*^72^ and angiogenin^73^. Studies investigating the effect of immune factors on microbiome composition, however, are more limited. A study in zebrafish has demonstrated that the host plays an important role in selecting its particular biota^74^. Furthermore, studies in mice with defensin deficiency *(Mmp7*^*−/−*^) or surplus (*Hd5*^*+/+*^) have shown a defensin-dependent reciprocal shift in the dominant bacterial species of the small intestine, without changes in total bacterial numbers^75^. Our findings did not identify significant differences in microbiota composition of cecal content as well as ileal content from *mir-802* null mice. We believe this can be best explained by the topology of *miR-802* expression and action, which is restricted to the upper small intestine where microbiota mass is small. Furthermore, our microbiota studies also indicate that the phenotypic effects in *mir-802* knockout mice are largely unrelated to the microbiota.

We investigated the *miR-802–Tmed9* axis using a knock-in model in which the *miR-802* sites in the *3*′*UTR* of *Tmed9* are selectively mutated, leading to selective derepression of *Tmed9* in the intestine. These mice showed a partial Paneth cell phenocopy of mice lacking *mir-802*, with increased *Lyz* and defensin expression. However, the antimicrobial phenotype was more pronounced in *Vil-Cre mir-802*^*fl/fl*^ mice, indicating that this pathway is also regulated by additional miR-802 targets. This may also explain why stem cell proliferation and Paneth cell numbers were not changed in*Vil-Cre Tmed9KI*^*mut/mut*^ mice and why no protective effect against Salmonella infection was observed. In addition, increased ROS was only seen in *Vil-Cre mir-802*^*fl/fl*^ mice, suggesting that ROS metabolism is regulated by many direct (*Nox1*) and indirect targets (antioxidant genes), independent of Tmed9.

Lastly, a previous study reported that *miR-802* regulates glucose homeostasis in the liver through a mechanism involving the repression of the transcription factor *Hnf1b*^8^. We were unable to detect differences in *Hnf1b* and glucose levels or insulin tolerance of mice with liver-specific deletion of *miR-802* under chow or high-fat diet conditions. Our data rather indicates that *miR-802* influences glucose metabolism through regulating glucose uptake and intestinal homeostasis.

In conclusion, we have identified and genetically validated a previously unknown regulatory miRNA network that is responsible for maintaining normal intestinal epithelial homeostasis and linking *miR-802*, TMED9, and WNT signaling. Considering the importance of this pathway in inflammatory bowel disease (IBD) and cancer it will be important to investigate the pathophysiological role of *miR-802* in initiation, remission, and relapse of IBD as well as in the context of epithelial tumor formation and progression.

## Methods

### Animal husbandry and mouse strains

All mice were on a pure *C57BL/6N* background. Mice were housed in a pathogen-free animal facility at the Institute of Molecular Health Sciences at ETH Zurich. The animals were maintained in a temperature- and humidity-controlled room on a 12 h light/12 h dark cycle (lights on from 6 am to 6 pm). Mice were fed a standard laboratory chow diet and had access to water ad libitum. Mice were 6–10 weeks of age unless indicated otherwise in the figures and text. All ethical regulations were complied with and all animal experiments were approved by the Kantonale Veterinäramt Zürich. *Vil-Cre* mice (B6.SJL-Tg(Vil-cre)997Gum/J), were purchased from Jackson Laboratories and *Lgr5-Cre* (B6.129P2Lgr5tm1(cre/ERT2) Cle/J) mice were a gift from H. Clevers.

### Generation of conditional miR-802 knockout mice

*miR-802* is an intergenic miRNA located on mouse chromosome 16 (chr16: 93369720-93369816+), and it was targeted through the introduction of loxP sites (34 bps) at positions chr16: 93369146 (575 bp upstream of miR-802) and chr16: 93370283 (468 bp downstream of *mir-802*). The 15.9 kb length targeting construct harboring mmu-miR-802 (pArnie/1480ABC A08) was generated by PCR cloning using a BAC template. LoxP sites were placed 608 bps upstream and 2287 bps downstream of miR-802. The neomycin cassette with FRT sites was inserted right before the second loxP site 1046 bp downstream of *mir-802*. All sequences were validated by DNA sequencing. The targeting vector was electroporated into C57BL/6 embryonic stem (ES) cells and single clones, selected for neomycin resistance, were picked and screened for homologous recombination by Southern blotting. Subsequently, correctly targeted ES cells were injected into blastocysts and transplanted into pseudopregnant females. The resulting chimeric offspring were bred to FLP-deleter mice for excision of the neomycin excision cassette and genotyped by Southern blotting. For tissue-specific *miR-802* ablation, the following strains were utilized: *Vil-Cre* (for intestinal epithelium-specific deletion), and *Lgr5-Cre* to genetically mark the Lgr5 positive stem cell population.

### Generation of conditional *Tmed9* 3′UTR mutant knock-in (*Tmed9KI*^*mu*t^) mice

The BAC clones RP23-62D23 and RP23-216P14 from the C57BL/6J library containing the mouse *Tmed9* gene (NCBI Reference Sequence: NM_026211.3) were used as a template to generate the homology arms by high fidelity PCR and sequencing. In the targeting vector, the Neo cassette was flanked by Rox sites^76^. A targeting vector was generated in which the mutant exon 5, flanked by lox2272 sites^77^ and containing two GTTACTG to GTATGTG mutations, was cloned downstream of the wild-type exon 5, containing the TAG stop codon and 3′UTR, in the reverse orientation. Wild-type and mutant exon 5 were flanked with loxP sites. After confirming correctly targeted ES clones via Southern Blotting, some clones were selected for blastocyst microinjection, followed by chimera production. Founders were confirmed as germline-transmitted via cross-breeding with wild-type mice. For intestinal epithelium-specific deletion the *Tmed9KI*^*mut/mut*^ mice were crossed with the *Villin-Cre* (B6.SJL-Tg(Vil-Cre)997Gum/J).

### Metabolic experiments

For intraperitoneal glucose tolerance tests (iPGTT), female or male mice were fasted for 6 h and then injected with 2 g/kg body weight D-glucose in PBS. In oral glucose tolerance tests (oGTT), glucose was orally administered by gavage. Blood glucose was measured using a Contour glucometer (Bayer) at specific time points. For insulin tolerance tests, mice were intraperitoneally injected with 0.4 U/kg insulin solution (Insulin solution human, Sigma Aldrich) and blood glucose was measured at the time points indicated in figures.

### In vivo GLP-1 secretion and GLP-1 content assays

In GLP-1 secretion assays, female mice were injected intraperitoneally with dipeptidyl peptidase-4 (DPP-4) inhibitor Sitagliptin (Merck, 3 mg/kg) at *t* = 0 and after 30 min, with D-glucose (Sigma Aldrich 3 g/kg). Blood was sampled 5 min thereafter and added to 5 μl Aprotinin (Sigma, 5 mg/ml), 2 μl EDTA (0.5 M) and, 3 μl DPP-IV Inhibitor (Millipore) on ice. The serum was collected and GLP-1 content was measured with GLP-1 ELISA (Merck).

### Glucose uptake assay

Female mice were orally administered a radiolabeled glucose solution through oral gavage. Unlabeled 40% glucose was combined with 370 Bq/µl [^14^C(U)]-D-glucose (50 µCi/1.85 MBq; PerkinElmer) and with 370 Bq/µl [1-^3^H(N)]-D-Mannitol (Perkin Elmer) (2 µCi), the latter necessary for correct glucose uptake into tissue for the adherent extracellular fluid phase^78^. Mice were challenged with glucose at 4 g/kg. After 15 min, blood was collected and mice were euthanized in a CO_2_ chamber. Blood was centrifuged at 1200 × *g* for 20 min before ^14^C tracer contents in plasma were measured in a liquid scintillation counter. For intestinal glucose uptake analysis, the whole small intestine was longitudinally opened and washed in ice-cold Krebs buffer for 10 min (119 mM NaCl, 4,7 mM KCl, 2,5 mM CaCl_2_, 1.2 mM MgSO_4_, 1.2 mM KH_2_PO_4_, 25 mM NaHCO_3_, pH 7.4). Tissues were then solubilized in Soluene (Perkin Elmer) and radioactivity was measured with a liquid scintillation counter. Background counts from nonradioactive control samples were subtracted from each sample.

### In vivo glucose-stimulated insulin secretion assay

Female mice were orally administered with 2 g/kg D-glucose. 15 and 30 min later, blood samples were collected and plasma insulin concentrations were determined by rat insulin enzyme-linked immunosorbent assay (ALPCO, Salem NH).

### Enterocyte and Crypt isolation

Enterocytes were isolated from the duodenum (2 cm), upper jejunum (referring to the proximal half of jejunum)), lower jejunum (distal half of the jejunum), and ileum (2 cm segment before cecum) as well as the colon of female 6–8 week old mice using a solution of 5 mM EDTA/PBS, supplemented with Complete protease inhibitor mixture (Roche) (40 mm) as published previously^79^. For the cell separation process, tissues were rotated in this solution for 1 h at 40 rpm. Cells were pelleted by sequential centrifugation steps after removal of tissue and resuspended immediately either with Trizol (Sigma) for RNA extraction or RIPA lysis buffer for protein extraction.

Upper jejunal crypts were isolated by using a previously described protocol^80^. In short, small intestines were first flushed with PBS, then cut longitudinally. The tissue was chopped into 4–5 mm pieces, and further mechanically sheared in PBS. The tissue fragments were rotated for 20 min in a 2 mM EDTA-PBS solution followed by mechanical shearing with a 10 ml pipette. After many suspension and centrifugation steps, the supernatant fractions enriched for crypts were passed through a 70-micron strainer (BD Biosciences) to remove the remaining cells of the intestinal villi fraction. The filtered fractions were then centrifuged at 150–200 × *g* for 3 min to collect the crypt fraction.

### RNA isolation and quantification

RNA was isolated using TRIzol reagent (Invitrogen) according to the manufacturer’s protocol. RNA was subjected to DNase I treatment with the DNA-free kit (Invitrogen), when necessary. RNA was reverse transcribed using the High Capacity cDNA Reverse Transcription Kit (Applied Biosystems). Quantitative PCR (qRT-PCR) was performed in an LC480 II Lightcycler (Roche) and using gene-specific primers and either Light Cycler 480 SYBR Green Master mix (Roche) or Sybr Fast 2x Universal Master mix (Kapa). Results were normalized to 36b4 or β-Actin mRNA levels. Sequences of primers are shown in Supplementary Data 1. Levels of miRNAs were measured using the TaqMan microRNA assays (Applied Biosystems) and the results were normalized to miR-16 or sno-202 levels. To recover RNA from pico-scale samples, the Picopure RNA isolation kit was utilized according to the manufacturer’s protocol (Thermo-Fisher Scientific).

### Cell culture, transfection, and viral infections

C2BBe1(Clone of Caco-2) and HEK293T cells were purchased from ATCC. HEK293T cells were grown in Dulbecco’s modified Eagle’s Medium (DMEM) supplemented with 2 mM L-glutamine, 10% fetal bovine serum (FBS), 100 U ml^−1^ penicillin, and 100 µg ml^−1^ streptomycin (all from Invitrogen) at 5% CO_2_ and 37 °C. C2BBe11 cells were grown in ATCC formulated Dulbecco’s Modified Eagle’s Medium supplemented with 0.01 mg/ml human transferrin (Sigma), 10% fetal bovine serum (FBS), 100 U /ml penicillin, and 100 μg/ml streptomycin (all from Invitrogen). All cells were transfected with either Lipofectamine 2000 (for plasmids) and Lipofectamine RNAimax (for siRNAs) (both from Life Technologies) according to the manufacturer’s protocol. siRNAs (Microsynth and Dharmacon) were transfected at 20–40 nM. Cells were harvested 24–48 h after transfection for further analysis. C2BBe1 cells were transduced with recombinant adenovirus at a multiplicity of infection (MOI) of 50. The medium was changed to a normal medium 6 h after transduction and cells were maintained for 36-48 h before harvesting.

### Luciferase assays

HEK-293 cells were cultured in 96-well plates and transfected with 20 ng pmirGLO reporters together with miRNA mimics (40 nM) or Adenovirus. Cells were harvested and assayed 48 h after transfection using the Dual-Luciferase Reporter Assay System (Promega). Results were normalized to the Renilla luciferase control contained in pmirGLO and expressed relative to the average value of the controls.

### Plasmids and recombinant adenoviruses

A mouse *miR-802*–expressing vector was generated by PCR amplification of a region consisting of the *miR-802* stem-loop, with an additional 142 and 156 bp of genomic sequence at the 5′ and 3′ ends, respectively. Fragments were cloned at BamHI and NotI sites of pVQAdCMV_K-NpA for adenovirus production (Viraquest). For generation of VQAd Tmed9-GFP (Ad-Tmed9), human *TMED9* cDNA was subcloned from a plasmid (Origene, MC200132, cDNA clone MGC: 7860) into pVQAdCMV_KNpA by restriction digestion with KpnI and XhoI (1429 bp insert). All adenoviruses harbor a GFP gene with an independent (RSV) promoter as a control for infection efficiency. Ad-Ctrl was based on the same vector backbone (including GFP) but lacked the transgenes. Wnt3a-FLAG plasmid was obtained from Origene, and M50 Super 8x TOPFlash (WT, #12456) and M51 Super 8x FOPFlash (TOPFlash mutant, #12457) plasmids were obtained from Addgene.

Mouse 3′ UTR sequences of putative miR-802 target genes were PCR-amplified from mouse DNA and cloned into pmirGLO vectors. Optimal amplicon size was elaborated by testing three different constructs in MIN6 cells by transfection (including an additional 50, 150, or 250 bp upstream and downstream of the *miR-802* stem-loop) and evaluating *miR-802* expression levels. Mutagenesis of the *miR-802* seed motifs in the 3′ UTR of selected targets was performed with QuickChange Lightning kit (Agilent Technologies). All generated constructs were validated by DNA sequencing.

### Immunoblotting

Lysates of mouse tissues or cells were homogenized in RIPA-Lysis buffer (150 mM NaCl, 50 mM TrisHCl pH 8, 5 mM EDTA pH 8, 5 mM EGTA pH 8, 1% NP-40, 0.5% Sodium deoxycholate, 0.1% SDS (Sigma-Aldrich) containing EDTA-free protease inhibitor cocktail (Roche Diagnostics) (1 tablet/50 ml). Cultured cells were homogenized by scraping and repeated pipetting, while tissues were disrupted using stainless steel beads and a Tissue-Lyser II (Qiagen). For analysis of secreted proteins, cell media were collected and concentrated using Amicon centrifugal filters. Supernatants were transferred to a new tube prior to determining the protein concentration using the bicinchoninic acid (BCA) kit (Sigma-Aldrich). After adding Laemmli buffer, lysates were boiled for 5 min at 95 °C and proteins were separated by sodium dodecyl sulfate-polyacrylamide gel electrophoresis (SDS-PAGE), and transferred onto nitrocellulose membranes by electroblotting in a wet chamber (Bio-Rad Laboratories). The membranes were stained with Ponceau S and blocked with 5% non-fat dry milk TBS-0.1%Tween (Sigma-Aldrich). The blots were incubated with primary antibodies overnight at 4 °C and developed using horseradish peroxidase-conjugated secondary antibodies (Calbiochem) followed by chemi-luminescent detection with a Fujifilm analyzer (LAS-4000). Uncropped blots are found in the Source data file.

### Antibodies

The antibodies used for immunoblotting are listed in Supplementary Table 1.

### Flow cytometry and isolation of ISCs, Paneth cells, and CD24+^LOW^ enriched stem cell fraction

Crypts and enterocytes were isolated from the upper jejunum (6 cm) of the small intestine of female mice as described previously in the methods. ISC and Paneth cells were isolated from the small intestine by flow cytometry following the protocol previously published^81^. In short, the crypt suspensions were centrifuged for 5 min at 250 × *g* (4 °C or room temperature). The pellets were resuspended in 1.0 ml of undiluted TrypLE Express (Invitrogen) + 120 μl of DNase I (10 U/μl, Roche) and transferred to 15 ml falcon tubes. Samples were incubated at 32 °C in a water bath for 2 min. Then, 12 ml cold DMEM (Invitrogen) were added to the samples and triturated twice, followed by centrifugation at 250 × *g* at 4 °C. Both enterocytes and crypt cells were then stained in cell staining buffer (Biolegend) with an antibody cocktail containing CD45-PE (eBioscience, 30-F11), CD31-PE (Biolegend, Mec13.3), Ter119-PE (Biolegend, Ter119), CD24-Pacific Blue (Biolegend, M1/69) and EPCAM-APC (17-5791-82, ThermoFisher Scientific) for 30 min at 4 °C in the dark. After that cells were washed twice with PBS and stained in a cell staining Buffer with Efluor-780 Viability dye (ThermoFisher Scientific, 65-0865-14) for 15 min. After washing cells twice, the pellets were dissolved in 2 ml FACS Buffer (2% FBS-PBS) and samples were filtered through 40 μm mesh (BD Falcon) and were further proceeded for sorting. Intestinal stem cells were isolated as Lgr5-EGFP^+^ Epcam^+^ CD24^low^/CD31^−^ Ter119^−^ CD45^–^Efluoro780^− ^^81^ Paneth cells were isolated as CD24^hi^SideScatter^hi^Lgr5-EGFP^−^EPCAM^+^/ CD31^−^Ter119^−^ CD45^−^Efluoro780^−^, enriched enterocyte population was isolated as CD24^–^ Lgr5-EGFP^–^EPCAM^+^/ CD31^−^Ter119^−^CD45^–^Efluoro780^−^ with a BD FACS Aria II SORP cell sorter. Enriched stem cell fraction was sorted as live single CD45^−^ CD24^+,LOW^ as previously shown^82^. Cells were incubated with both CD24-PE (BD 553262) and CD45-FITC (BD 553080) antibodies, at 0.25 μg per 1 × 10^6^ cells in 100 μl PBS/1% BSA, for 15-min RT, and washed with PBS/1% BSA. PicoPure RNA isolation kit was used for RNA isolation.

### Illumina RNA sequencing and analysis

RNA was isolated from enterocytes of 14-week-old female mice using Trizol (Sigma), followed by RNeasy (Qiagen) column purification with on-column DNase (Sigma) digestion. The quality of isolated RNA was determined with a Qubit (1.0) Fluorometer (Life Technologies) and a Bioanalyzer 2100 (Agilent). The TruSeq RNA Sample Prep Kit v2 (Illumina) was used according to the manufacturer protocol. Briefly, total RNA samples (100–1000 ng) were poly-A enriched and then reverse transcribed into double-strand cDNA. The cDNA samples were fragmented, end repaired, and polyadenylated before ligation of TruSeq adapters containing the index sequence for multiplex sequencing. Multiplexing fragments containing TruSeq adapters on both ends were selectively amplified with PCR. The quality and quantity of the enriched libraries were validated using Qubit (1.0) Fluorometer and the Caliper GX LabChip GX (Caliper Life Sciences Inc.). The product is a smear with an average fragment size of ~260 bp. The libraries were normalized to 10 nM in Tris-Cl 10 mM, pH 8.5, with 0.1% Tween 20. RNA-Seq read qualities were checked with FastQC (Babraham Bioinformatics), which computes various quality metrics for raw reads. Reads were aligned to the genome and transcriptome with TopHat v 1.3.3 (https://ccb.jhu.edu/software/tophat/manual.shtml). Before mapping, the low-quality ends of the reads were clipped (3 bases from the read start and 10 bases from the read end). TopHat was run with default options. The fragment length parameter was set to 100 bases, with an SD of 100 bases. Based on these alignments, the distribution of the reads across genomic features was analyzed. For seed enrichment analysis, only genes with FPKM above 1.0 were considered as targets. For cumulative distribution function calculations, log_2_ fold-change values were corrected for 3′ UTR length biases. To detect differentially expressed genes we applied a count-based negative binomial model implemented in the software package EdgeR (R version: 3.3.0, EdgeR version: 3.14.0) [EdgeR]. The differential expression was assessed using a glm adapted for over-dispersed data. Genes showing altered expression with adjusted (two-tailed Benjamini and Hochberg method) *p*-value < 0.05 were considered differentially expressed. Samples were sequenced at the Functional Genomic Center Zurich (FGCZ) using the Illumina HiSeq 2500.

### DNA extraction for 16S rRNA gene sequencing

Cecal and ileal content was collected from co-housed wildtype (*mir-802* ^*fl/fl*^) and *Vil-Cre mir-802* ^*fl/fl*^ mice fed conventional mouse chow. Isolated cecum and ileal content was immediately flash-frozen in liquid nitrogen and stored at −80 °C. DNA was extracted using the AllPrep DNA/RNA Kit (Qiagen) with the following changes in the disruption and homogenization steps: 600 mL of RLT buffer and two 3 mm metal beads were added to the tube containing the cecum content and bead-beaten at 10 Hz for 2 min using the mixer mill Retsch MM400. To separate fibers from bacteria, samples were centrifuged at 700 × *g* for 2 min. Supernatants containing the bacteria were transferred to a tube containing 0.9 mg of 0.1 mm zirconia beads (OPS Diagnostics) and samples were homogenized twice at 30 Hz for 3 min with 5 min incubation between each homogenization run. Before the transfer to DNA-binding columns, the samples were centrifuged at full speed for 3 min to pellet the cell debris. The supernatants were loaded onto the DNA-binding columns and DNA was eluted in 100 µL elution buffer (EB). A water sample as negative control and a sample with DNA from *E.coli* DH5α as positive control were processed in the same way as the cecum samples.

### Library preparation and 16S rRNA gene sequencing

The library was produced with a two-step PCR approach using the amplicon deep sequencing primers from Microsynth. The first-step PCR used the locus-specific degenerative primers 515F (5′-GTG**Y**CAGCMGCCGCGGTAA-3′) and 806rB (5′-GGACTAC**N**VGGGTWTCTAAT-3′) targeting the variable region 4^83,84^. The PCR reaction was performed using Q5 High-Fidelity DNA polymerase (BioConcept, New England BioLabs) under the following cycle conditions: 1. initial denaturation, 95 °C for 4 min; 2. denaturation, 95 °C for 30 s; 3. annealing, 56 °C for 30 s; 4. extension, 72 °C for 30 s; 5. final extension, 72 °C for 4 min. Cycles 2–4 were repeated eight times for the first PCR reaction and twenty times for the second PCR reaction. After each PCR reaction, the PCR products were cleaned from oligos and nucleotides using 0.8 × reaction volumes of CleanNGS magnetic beads (LABGENE SCIENTIFIC SA). The cleaned PCR product was eluted in 14 µL of EB and used in a second PCR reaction with barcoded primers or for producing multiplexed samples. The quantity of amplicons was measured using a Qubit 4 fluorometer (Thermo Fisher Scientific). The quality of the amplicons was verified using a fragment analyzer (Advanced Analytical). The length of the amplicons after barcoding was ~450 bp. PCR products were adjusted to a final concentration of 60 ng DNA per 20 µL of multiplexed samples. Paired-end read sequencing was performed on the Illumina MiSeq platform at the Functional Genomics Center, Zürich.

### Analysis of microbiota composition

The raw sequencing data were processed using USEARCH (version 11.0.667)^85^. Paired reads were merged and quality filtered using the *fastq_mergepairs* command with default settings. Merged reads were filtered using the *fastq_filter* command (*-fastq_maxee 0.1*) and only merged reads with perfect primer matches and a minimum length of 100 bp were selected. Sequences were de-replicated using the *fastx_uniques* command and clustered into zero-radius zOTUs using the *unoise3* command, which also removed chimeric sequences^86^. zOTU abundances for each sample were quantified using the *otutab* command (*-strand both; -id 0.97*). Taxonomic annotation was performed by querying zOTU sequences against the SILVA database (version 128) using the *usearch_global* command (*-id 0.90; -maxaccepts 20; -maxrejects 500; -strand both; -top_hits_only*). The entire pipeline is available at github.com/SushiLab/Amplicon_Recipes. Further analyses were performed in RStudio (version 1.2.1335 based on R version 3.6.3) using the libraries *vegan*, *ggplot2,* and *DESeq2*. We analyzed a total of 1.1 × 10^8^ sequencing reads: 1.0 × 10^7^ reads for the cecum and ileum samples, 6.8 × 10^3^ for water and EB-buffer (negative control), and 5.7 × 10^5^ reads for a microbial community standard (positive control (Zymo Research D6311)). For analyses based on relative abundances, read counts were rarefied to 128,263 reads per sample using the *rarefy* function.

For principal component analysis, Euclidean distances after Hellinger transformation were computed between samples using the *vegdist* function. Differentially abundant zOTUs between samples from wild-type and *Vil-Cre mir-802*^*fl/fl*^ mice were identified using raw count data and the DESeq2 R package after accounting for the cage effect (i.e., using the cage as a grouping variable in the test design).

### Data analysis

RNA-Seq read qualities were checked with FastQC (Babraham Bioinformatics), which computes various quality metrics for raw reads. Reads were aligned to the genome and transcriptome with TopHat v 1.3.3 (https://ccb.jhu.edu/software/tophat/manual.shtml). Before mapping, the low-quality ends of the reads were clipped (3 bases from the read start and 10 bases from the read end). TopHat was run with default options. The fragment length parameter was set to 100 bases, with an SD of 100 bases. Based on these alignments, the distribution of the reads across genomic features was analyzed. For seed enrichment analysis, only genes with FPKM above 1.0 were considered as targets. For cumulative distribution function calculations, log2 fold-change values were corrected for 3′ UTR length biases.

### Histopathological examination and immunofluorescence

Small intestines were fixed in 4% paraformaldehyde (PFA) followed by vertical embedding in paraffin. Sections were cut from paraffin-embedded tissues at a 3.5 μm thickness, deparaffinized, and antigen retrieved by boiling them in Citrate buffer (pH 6). The sections were permeabilized and blocked in PBS solution with 0.1% Triton X-100, 1% BSA, and 5% donkey or goat serum depending on the antigen host. Sections were stained with primary antibodies overnight at 4 °C, followed by secondary antibody incubation at room temperature for 1 h.

The lysozyme-positive cells in the crypt were quantified and normalized to DAPI-positive cells in crypt (60 crypt for each mouse) using NIH ImageJ software (http://rsbweb.nih.gov/ij/download) and normalized to crypt number. For intestinal histology analysis, sections were cut at 3.5 μm. A total of 5–6 sections, at least 400 μm apart, were cut from each mouse and further processed with routine H&E. All small intestinal sections were scanned using a ×20 objective of the Pannoramic 250 Slide scanner (3D Histech) or by Confocal microscopy. Villous height and crypt depth were measured in at least 30 well-oriented, villus-crypt units per mouse using ImageJ and CaseViewer.

### Alcian Blue staining

For Alcian Blue staining, the intestinal tissues were fixed in Carnoy’s solution (60% ethanol, 30% chloroform, and 10% glacial acetic acid) for 4 h at room temperature. Subsequently, the tissues were transferred in 100% ethanol overnight and further processed for paraffin sectioning. Following deparaffinization, slides were stained in Alcian blue solution (3%, pH 2.5, Sigma) for 30 min. Slides were washed in tap water for 2 min, counterstained with nuclear fast red solution (Sigma-Aldrich) for 5 min, and followed by dehydration in ethanol gradient solution. Goblet cells were quantified using the Cell counter ImageJ software and normalized to crypt numbers.

### FITC intestinal permeability

Female mice received FITC by oral gavage at a concentration of 60 mg/kg body weight after 6 h of water fasting. After 4 h the blood was collected and centrifuged for plasma separation. The plasma was diluted 1:1 in PBS and FITC concentration was determined by Tecan Fluorescence with an excitation of 485 nm and emission wavelength of 528 nm.

### ROS assay

For ROS measurement the kit from MyBioSource (MBS723353) was used. Briefly, proximal jejunum was washed in ice cold PBS and sonicated on ice in the buffer provided. The samples were subsequently centrifuged and assayed immediately following the manufacturer’s protocol. Data were normalized to protein concentration.

### iNOS Elisa

For iNOS measurement in the jejunal intestine the kit from Lubioscience (STA-347) was used. Briefly, tissues were resuspended in 1 ml PBS and sonicated on ice. The samples were subsequently centrifuged at 10,000 × *g* for 5 min and assayed immediately following the manufacturer’s protocol.

### Intestinal epithelial cell proliferation and turnover

For assessing intestinal epithelial cell proliferation, female mice were injected with Bromo-2′-deoxyuridine (BrdU) in PBS intraperitoneally (100 µg/g body weight) and euthanized 1.5 h later. BrdU-labeled cells in the jejunum were detected using BrdU antibody. The BrdU-positive cells in the crypt were quantified and normalized to crypt number (60 crypt per mouse) using NIH ImageJ software. For analyzing epithelial cell turnover, BrdU in PBS was injected at the same dose. After 36 h, mice were sacrificed and BrdU-positive cells were detected using BrdU antibody.

### EDU flow cytometry

Female mice were injected with 20 mg/kg of body weight EDU in PBS. After 1 h incubation mice were euthanized and crypts were isolated as previously described. Crypts were dissociated in single cells using TRIPLE enzyme for 2 min at 37 °C. After, cells were fixed and incubated with anti-EDU antibody following the kit instruction protocol of Click-iT® EdU Flow Cytometry Assay Kits. Cells were counterstained with Hoechst and EDU/positive single cells were measured using Flow cytometry BD LSRFortessa.

### TUNEL assay

For Tunel assay a commercially available kit Click-iT® TUNEL Alexa Fluor® Imaging Assay was used with some modifications. For this, freshly cut paraffin blocks were deparaffinized and rehydrated in alcohol gradients and fixed in paraffin for 15 min at RT. Subsequently, slides were digested in ProteinaseK for 15–20 min and fixed again for 5 min in 4% PFA. After this step, the normal kit instruction protocol was followed and slides were counterstained with Hoechst for the nuclei.

### Mouse small intestine organoid culture and virus infection

Small intestinal organoids were established from isolated crypts of the proximal intestine from wild-type C57BL/6N mice, and cultured as described previously^87^. Organoids were passaged weekly at a 1:5 split ratio and refed with media every 2–3 days. The basal culture media (advanced DMEM/F12 supplemented with Pen/strep, 10 mmol HEPES, Glutamax) was supplemented with N-acetylcysteine, 1 × B27, 1 × N2, 50 ng/ml EGF, 1 µg/ml R-spondin, and 0.1 µg/mL Noggin. All growth factors were purchased from Peprotech. For virus infection experiments, organoids were harvested in Corning recovery solution and mechanically dissociated in Basal Media using a narrowed Pasteur Pipette for 5–7 times up and down. After, organoids were centrifuged at 250 × *g* and resuspended in 400 ul full growth medium containing 100 × 10^5^ MOi virus for 100 µl matrigel. The mixture was incubated at 37 °C for 5 h, with continuous mixing every 30 min. Next, the mixture was centrifuged at 250 × *g* for 5 min and organoids were seeded in matrigel and cultured for 3 days before harvesting for RNA isolation.

### Cell fractionation

Crypts were isolated and the cell pellet was resuspended in Hypotonic Lysis Buffer (HLB, 10 mM HEPES, 1.5 mM MgCl_2_, 10 mM KCl, 1 mM DTT, and fresh protease inhibitor) and incubated on ice for 30 min. 6 μL 10% NP40 per 100 μL HLB (100 μl of HLB for 1 M cells) was added after the incubation and pipetting up and down.

An aliquot was taken as input and then spun down at 13 K rpm 35 s at 4 °C. The supernatant was saved as a cytosolic extract. Optionally, the nuclei were then washed in HLB to remove any contaminating cytoplasm and re-sedimented. The nuclei were then lysed in 20 μl Nuclear Lysis Buffer (NLB, 10 Mm HEPES, 100 mM KCl, 3 mM MgCl_2_, 0.1 mM EDTA, 1 mM DTT, and fresh protease inhibitor) and incubated on ice for 30 min. Extra 1/10th volume of 4 M (NH4)_2_SO_4_ was added over 30 min. After the lysate was spined at 13 K rpm 15 min at 4 °C, the supernatant was saved as the nuclear extract. The nuclear fraction, cytoplasmic fraction, and input were loaded in Laemmli buffer in 12% SDS gel, and membranes were incubated in H3 (CST 4499), and β-CATENIN (CST 8814) antibodies overnight at 4 °C.

### TopFLASH assay

The TOPFlash assay was performed in 96-well plates. For each well, cells were transfected with 0.15 μg TOPFlash (or FOPFlash with mutant TCF binding sites) and 0.02 μg pRL-SV40P using Lipofectamine 2000. For co-transduction with other plasmids, 6 h before transfection cells were transduced with 50 MOI Ad-miR-802 or Ad-Tmed9. For *Tmed9* knockdown experiments 20 nm si-Tmed9 were transfected with RNAimax 6 h before transfection. The luciferase activity was measured 24 h later using the Dual-Glo Luciferase Assay System (E2940; Promega) according to the manufacturer’s protocol. The TOPFlash or FOPFlash activity was normalized to Renilla luciferase signals. For transduction experiments, data were normalized to the Ad-ctrl treated samples with the equivalent MOI. For siRNA transfection experiments, data were normalized to the si-Ctrl treated samples with the same concentration (20 nm).

### *Salmonella* Typhimurium colonization assay

For the mono-colonization experiments, female, age-matched, littermate controlled, specified pathogen-free mice were orally gavaged with 10^8^ CFU of *Salmonella* Typhimurium strain SL1344^88^. The bacterial culture was initially grown in LB medium containing 0.3 M NaCl for 12 h at 37 °C and then subcultured for 4 h in fresh medium at a 1:20 dilution. The bacteria were spun down and resuspended in an equal volume of PBS prior to infection. Mice were sacrificed after 48 h and intestinal tissues were snap-frozen for RNA extraction. Small intestinal colonization levels were measured by dilution plating of luminal contents. Bacterial levels in spleen, feces, and mLN were determined by dilution plating of homogenized tissue.

### Reporting summary

Further information on research design is available in the Nature Research Reporting Summary linked to this article.

## Supplementary information

Supplementary Information

Description of Additional Supplementary Files

Supplementary Data 1

Reporting Summary

## Data Availability

Sequencing data have been deposited in the ArrayExpress under accession code: GSE148198, https://www.ncbi.nlm.nih.gov/geo/query/acc.cgi?acc=GSM4456008. Microbiome sequencing data have been deposited in European Nucleotide Archive (ENA) database under accession code: PRJEB43950. The authors declare that the data supporting the findings of this study are available within the paper and its supplementary information files. All remaining data will be available from the corresponding author upon reasonable request. Source data are provided with this paper.

## Source data

Source Data
