# Supplementary material for: miR-802 regulates Paneth cell function and enterocyte differentiation in the mouse small intestine
Source: Nat Commun. 2021 Jun 7;12:3339. doi: 10.1038/s41467-021-23298-3 (PMC8184787; doi:10.1038/s41467-021-23298-3)
Supplement: Supplementary file 1 — Supplementary Information [file 41467_2021_23298_MOESM1_ESM.pdf]

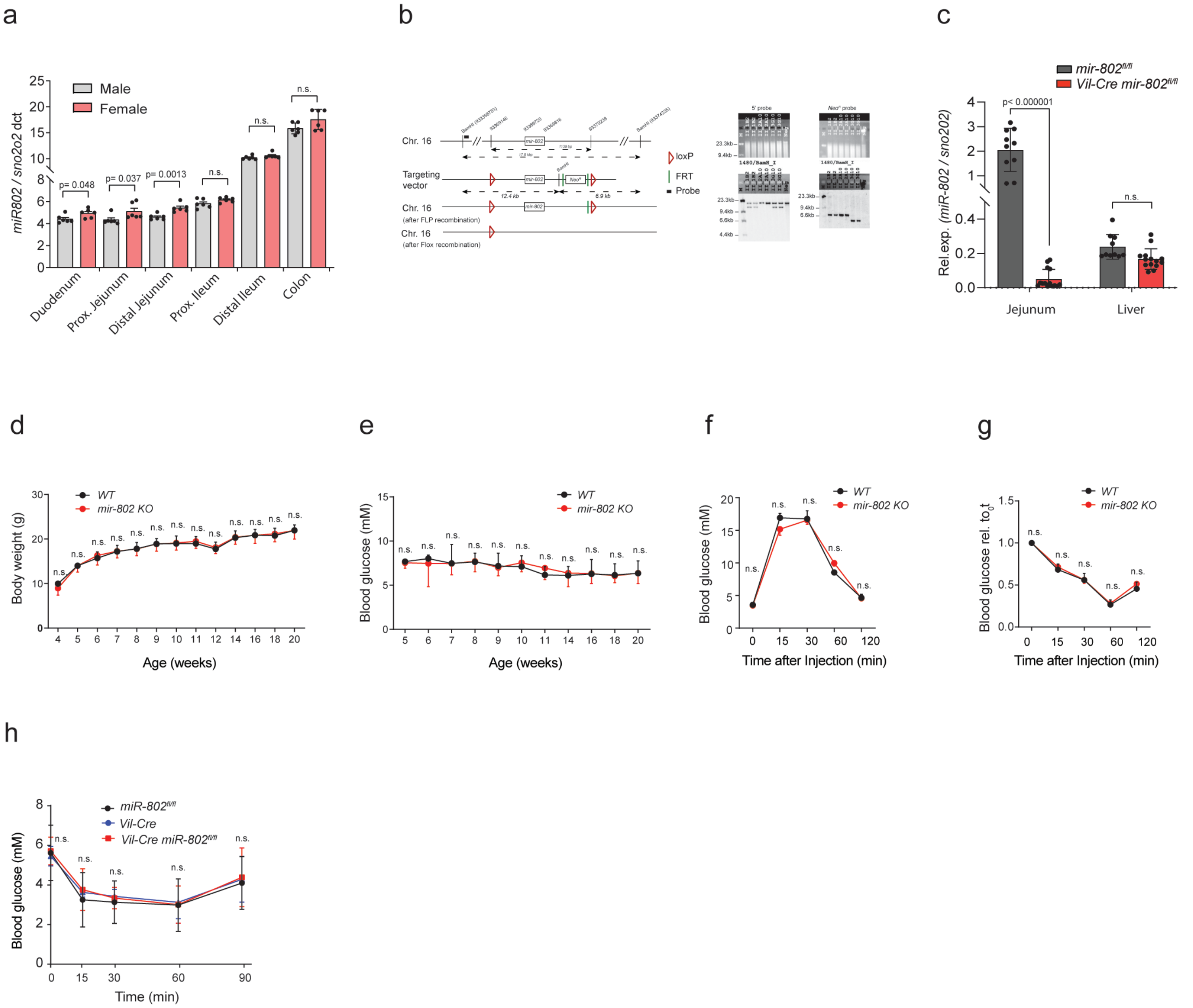

Supplementary Fig. 1 Generation and metabolic characterization of global *mir-802 KO*, and of conditional *Vil-Cre mir-802$^{fl/fl}$* mice.

a Expression of *miR-802* normalized by *Sno2o2*, measured by qRT-PCR, in male and female mice of duodenum, jejunum, ileum and colon (n= 6 per group). **b** Left: Schematic illustration depicting the strategy for the generation of the conditional mutant *mir-802* allele. Right: Southern blot analysis from wildtype and mutant biopsies using a 5' and *neo^R* probe. Data shown is from one experiment. **c** Relative *miR-802* expression, measured by qRT-PCR, in jejunum and liver of *Vil-Cre mir-802$^{fl/fl}$* and control *mir-802$^{fl/fl}$* mice (n= 10,14 respectively, per group). **d,e** Body weight (d) and blood glucose (e) levels of *ad libitum* fed mice in Chow diet of *mir-802 KO* compared to *WT* control (n= 26,20 per genotype). **f** Intraperitoneal glucose tolerance test (IPGTT) of female *mir-802 KO* and *WT* littermate control mice (n=15,12). **g,h** Intraperitoneal insulin tolerance test (IPITT) in female *mir-802 KO* and *WT* control mice (g) and in *Vil-Cre mir-802$^{fl/fl}$* (h) compared to control *mir-802$^{fl/fl}$* and *Vil-Cre* (n=13,9 for (g) and n= 8, 6, 7 for (h) respectively per genotype).

Data are plotted as mean ± SD. Significance was evaluated by two-tailed t-test (a) two-tailed t-test with Holm- Sidak correction for multiple comparison (c), mixed Anova with Holm-Sidak correction for multiple comparison (d,e,g), and two-way ANOVA for repeated measures with Sidaks multiple comparisons test (f,h).

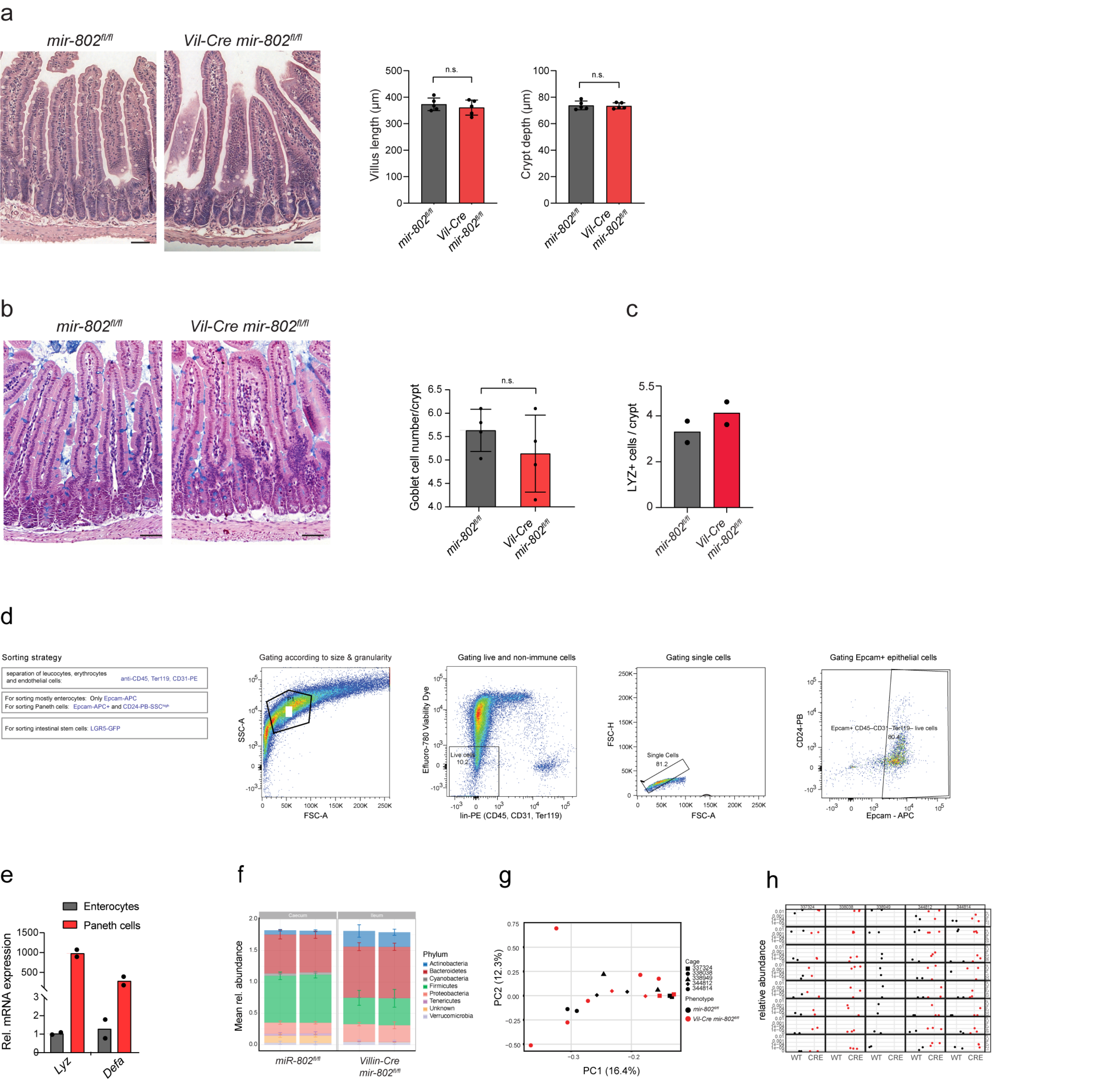

i

Differentially abundant zOTUs (family level annotation)	FDR corrected p-value
zOTU 27 (Lachnospiraceae)	0.027
zOTU 65 (Lachnospiraceae)	0.015
zOTU 336 (Ruminococcaceae)	0.015
zOTU 100 (Lachnospiraceae)	0.015
zOTU 243 (Lachnospiraceae)	0.02
zOTU 233 (Lachnospiraceae)	0.015
zOTU 348 (Lachnospiraceae)	0.015
zOTU 321 (Rhodospirillaceae)	0.015

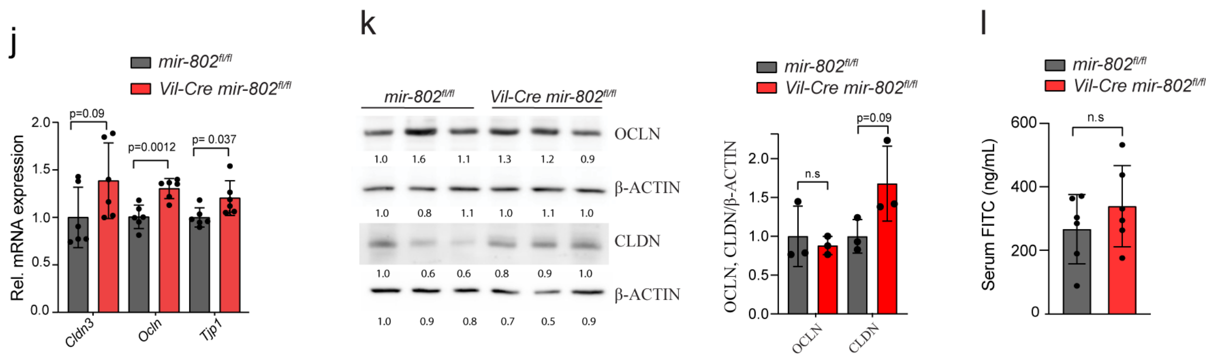

Supplementary Fig. 2 miR-802 regulates Paneth cell expansion

a Representative images of H&E staining (left) used to measure villi length and crypt depth (right) of jejunum from *Vil-Cre mir-802*$^{fl/fl}$ and *mir-802*$^{fl/fl}$ control mice. Villi length (≈80 villi/mouse) and crypt depth (≈60 open crypts/mouse) were analyzed using CaseViewer (n= 5 for each group). Scale bar: 40 µm. **b** Representative images of Alcian blue staining (left) of upper jejunal sections of mice with indicated genotypes and quantitative analysis of goblet cells (right) (n= 4, 4 per genotype). Scale bar: 40 µm. **c** Quantification of Paneth cell numbers/crypt in proximal ileum of *Vil-Cre mir-802*$^{fl/fl}$ and *mir-802*$^{fl/fl}$ mice. Each dot represents the average of at least 60 crypts per animal (n=2). **d** Schematic illustration of the sorting strategy and gating of the different intestinal cell populations. Gating according to size and granularity, live and non-immune cells, single cells, Epcam positive epithelial cells. **e** Relative expression of Paneth cells markers *Lyz* and *Defa* compared to enterocytes in sorted cells measured by qRT-PCR (n=2 per group). **f–i** 16S rRNA gene amplicon sequencing analysis of co-housed mice in five different cages. (f) Square root transformed mean relative phylum abundance with standard deviations for each phenotype showed no significant differences within the caecum and ileum samples. The colors indicate different bacterial phyla. (g) Principlal Component (PC) analysis was performed based on Euclidean distances of the Hellinger-transformed ileal zOTU abundance data. The symbols and colors denote cage and phenotype, respectively. The separation by cage is noticeable and suggests that co-housing had an impact on the cecal microbiota composition. (h) Relative abundance of individual zOTUs from caesum samples, which were significantly differentially abundant between phenotypes after accounting for cage effect as determined by DESeq2 analysis. For better visualization, a pseudo-count was added and set as the null reference, which indicates not detected zOTUs. (i) FDR corrected p-values for the eight zOTUs, which were significantly differentially abundant between the phenotypes (n= 19 for f–i). **j,k** Expression analysis of cellular adhesion genes by qRT-PCR (j) and immunoblotting (k) in jejunum of mice with indicated genotype (n= 6 per genotype for (j) and n= 3 for (k)). **l** Analysis of fluorescein isothiocyanate-conjugated (FITC) dextran in serum of *Vil-Cre mir-802*$^{fl/fl}$ and *mir-802*$^{fl/fl}$ mice following FITC oral gavage (n= 6 per genotype).

Data are plotted as mean ± SD. Significance was evaluated by two-tailed t-tests (a,b,j–l), two-sided Wilcoxon rank sum test (f), two-sided Permutational MANOVA with FDR correction to account for multiple comparisons (g), two-sided Wald-test with FDR correction accounting for multiple comparison (h,i).

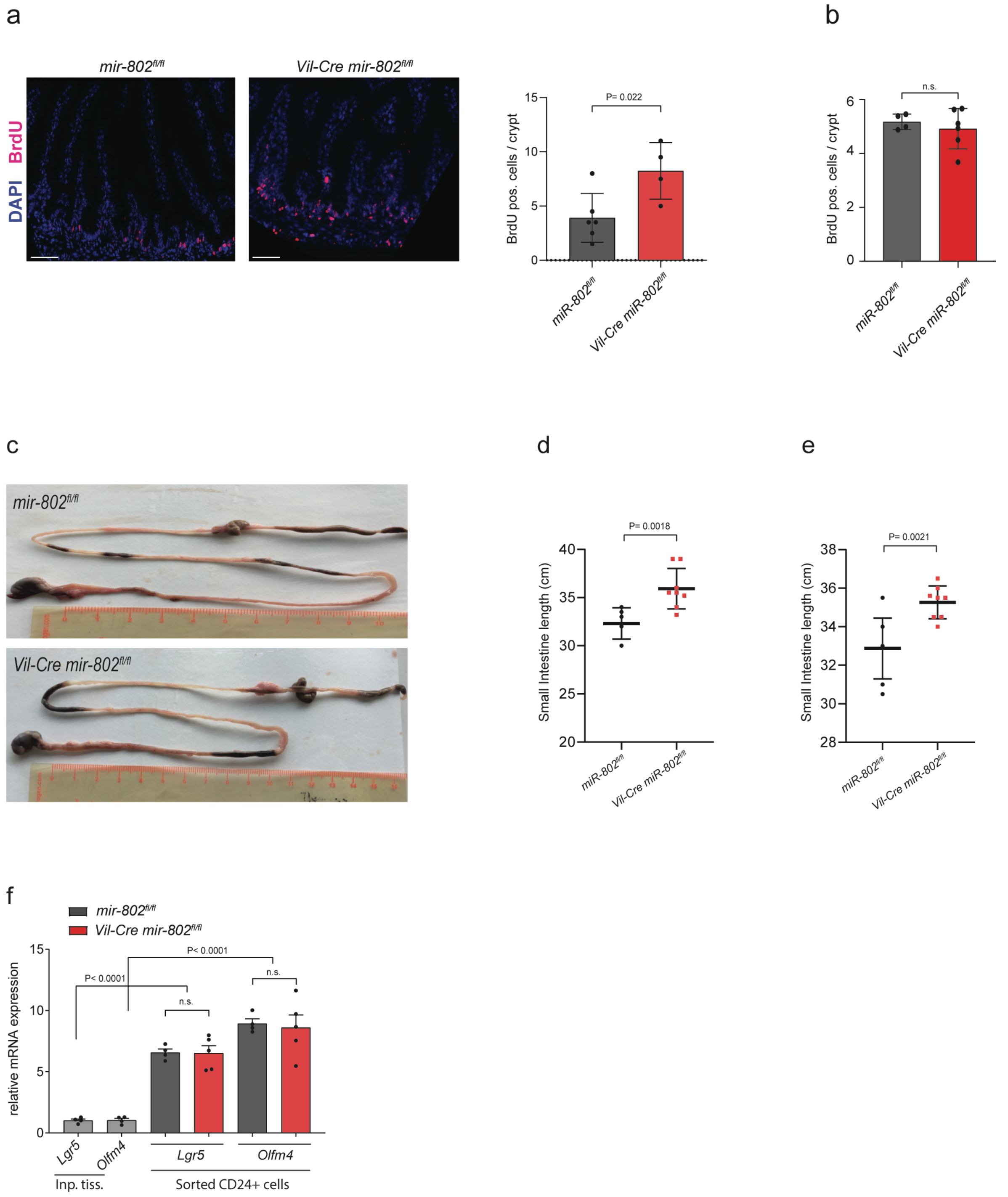

Supplementary Fig. 3. Increased intestinal proliferation in mice lacking *mir-802*.
a Representative immunohistological stainings of *Vil-Cre mir-802$^{fl/fl}$* and control *mir-802$^{fl/fl}$* newborn male mice that were injected with Brdu 90 min before sacrifice and stained with DAPI and anti BrdU antibody. Quantitative analysis of BrdU-positive cells per crypt is shown on the right. Scale bar: 50 µm (n= 4,6 respectively). **b** Quantification of BrdU-positive cells in ileal crypts of *Vil-Cre mir-802$^{fl/fl}$* and *mir-802$^{fl/fl}$* mice (n= 6, 4 per genotype). **c** Representative images of the intestine of male *Vil-Cre mir-802$^{fl/fl}$* and *mir-802$^{fl/fl}$* mice. **d,e** Quantitative analysis of gut length from 18 (d) and 28-week-old mice (e) (n= 8 per genotype). **f** Relative expression of stem cell markers *Lgr5* and *Olfm4* from sorted enriched stem cell fractions compared to input sample measured by qRT-PCR (n=4, 5 per genotype).
Data are plotted as mean ± SD. Significance was evaluated by two-tailed t tests (a,b,d,e), or one way Anova with Sidak's multiple comparison (f).

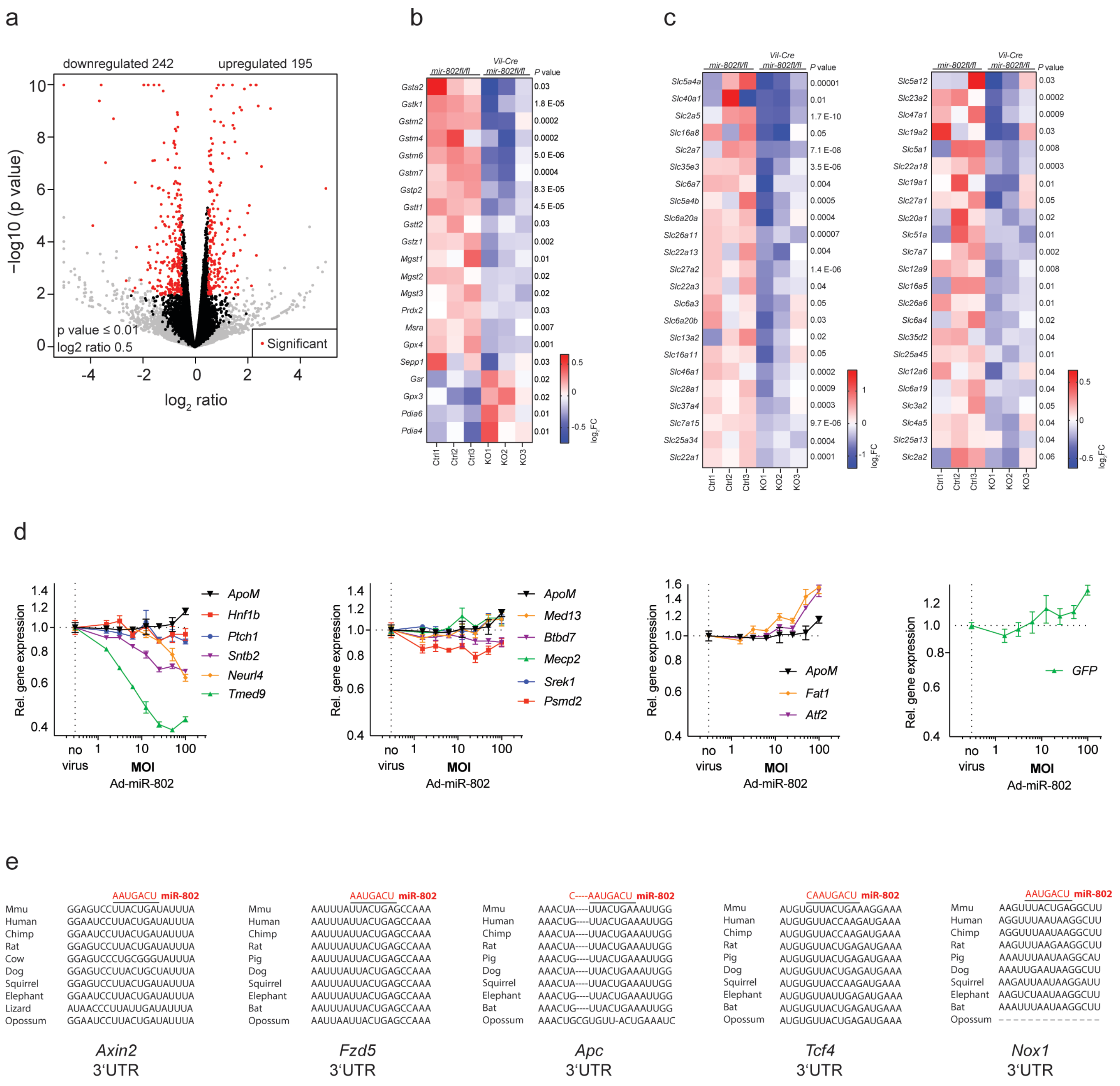

Supplementary Fig. 4 Transcriptome analysis of isolated intestinal epithelial cells from *Vil-Cre mir-802$^{fl/fl}$* and control mice. a Volcano plot from RNAseq data of the upper jejunum of *Vil-Cre mir-802$^{fl/fl}$* and control *mir-802$^{fl/fl}$* mice showing the downregulated and upregulated genes with with a cutoff of 0.5 log2 ratio (p-value 0.01). **b,c** Heatmap of transcripts from RNAseq data encoding antioxidant proteins (b) and solute carrier (c) of the upper jejunum of *Vil-Cre mir-802$^{fl/fl}$* and control *mir-802$^{fl/fl}$* mice shown as Log2FC (n= 3). **d** Primary hepatocytes derived from C57BL/6N mice transduced with different concentrations of adenovirus and analyzed for gene expression by qRT-PCR. Ad-miR-802 at MOI 0, 0.3, 1.6, 3.1, 6.3, 12.5, 25, 50 and 100. Gene expression is shownrelative to Ad-Ctrl or non-transduced cells. *ApoM* was analyzed as a control (non-target); all other genes contain conserved miR-802 target sites (n= 4 per condition). **e** Conservation of miR-802 seed sequences in 3'UTR of indicated species from *Axin2, Fzd5, Apc, Tcf4, and Nox1*. Data are expressed as mean ± s.e.m (d).

Figure S5

a

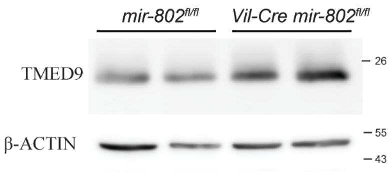

Supplementrary Fig. 5 TMED9 is regulated in jejunal *Vil-Cre mir-802$^{fl/fl}$* -derived organoids.

a Immunoblot of TMED9 protein from mouse small intestine organoids derived from *Vil-Cre mir-802$^{fl/fl}$* and control *mir-802$^{fl/fl}$*. β-Actin was used as a loading control. Each lane represents a lysate from a different mouse (n=2).

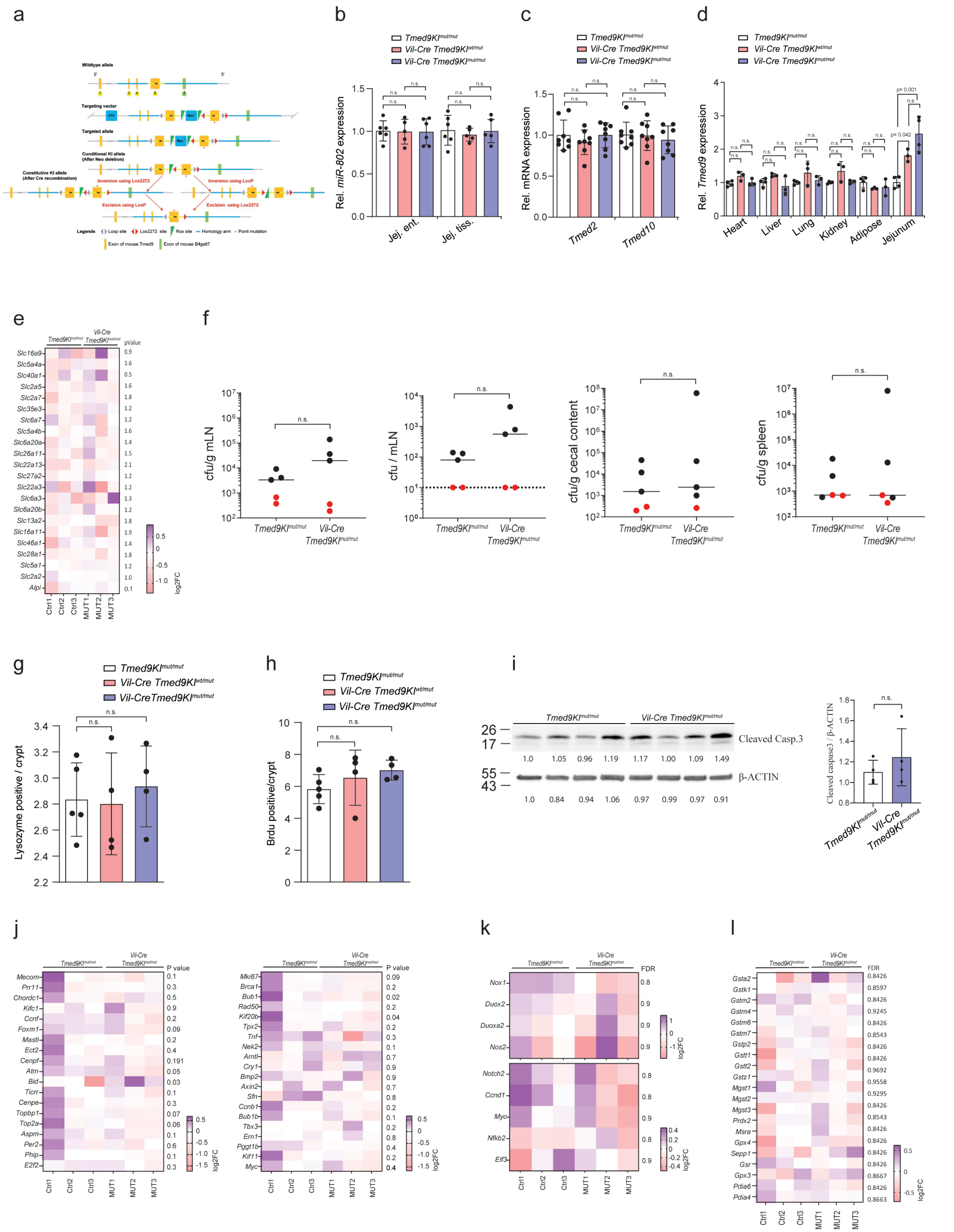

a
Wildtype allele
Targeting vector
Targeted allele
Conditional KI allele (After Neo deletion)
Constitutive KI allele (After Cre recombination)
Inversion using Lox2272
Inversion using LoxP
Excision using LoxP
Excision using Lox2272
Legends
Loxp site
Lox2272 site
Rox site
Homology arm
Point mutation
Exon of mouse Tmed9
Exon of mouse B4galt7
b
Tmed9KI mut/mut
Vil-Cre Tmed9KI wt/mut
Vil-Cre Tmed9KI mut/mut
Rel. miR-802 expression
Jej. ent.
Jej. tiss.
n.s.
c
Rel. mRNA expression
Tmed2
Tmed10
d
Rel. Tmed9 expression
Heart
Liver
Lung
Kidney
Adipose
Jejunum
p= 0.001
p= 0.042
e
pValue
log2FC
Slc16a9
Slc5a4a
Slc40a1
Slc2a5
Slc2a7
Slc35e3
Slc6a7
Slc5a4b
Slc6a20a
Slc26a11
Slc22a13
Slc27a2
Slc22a3
Slc6a3
Slc6a20b
Slc13a2
Slc16a11
Slc46a1
Slc28a1
Slc5a1
Slc2a2
Alpi
Ctrl1
Ctrl2
Ctrl3
MUT1
MUT2
MUT3
f
cfu/g mLN
cfu / mLN
cfu/g cecal content
cfu/g spleen
g
Lysozyme positive / crypt
h
Brdu positive/crypt
i
Cleaved Casp.3
β-ACTIN
1.0 1.05 0.96 1.19 1.17 1.00 1.09 1.49
1.0 0.84 0.94 1.06 0.97 0.99 0.97 0.91
Cleaved caspase3 / β-ACTIN
j
P value
Mecom
Prr11
Chordc1
Kifc1
Ccnf
Foxm1
Mastl
Ect2
Cenpf
Atm
Bid
Ticrr
Cenpe
Topbp1
Top2a
Aspm
Per2
Phip
E2f2
Mki67
Brca1
Bub1
Rad50
Kif20b
Tpx2
Tnf
Nek2
Arntl
Cry1
Bmp2
Axin2
Sfn
Ccnb1
Bub1b
Tbx3
Ern1
Pggt1b
Kif11
Myc
k
FDR
Nox1
Duox2
Duoxa2
Nos2
Notch2
Ccnd1
Myc
Nfkb2
Elf3
l
FDR
Gsta2
Gstk1
Gstm2
Gstm4
Gstm6
Gstm7
Gstp2
Gstt1
Gstt2
Gstz1
Mgst1
Mgst2
Mgst3
Prdx2
Msra
Gpx4
Sepp1
Gsr
Gpx3
Pdia6
Pdia4

Supplementary Fig. 6. Mutations in miR-802 binding sites of*Tmed9* partially phenocopy the *Vil-Cre mir-802$^{fl/fl}$* Paneth cell phenotype. **a** Illustration showing the targeting strategy for the generation of the conditional *Tmed9 mut* allele. **b** Relative *miR-802* levels, measured by RT-PCR, in jejunal isolated enterocytes and tissue of *Vil-Cre Tmed9KI$^{mut/mut}$*, *Vil-Cre Tmed9KI$^{wt/mut}$*, and control mice *Tmed9KI$^{mut/mut}$* mice (n= 6,5,6 respectively, per genotype). **c** Relative *Tmed2* and *Tmed10* mRNA levels, measured by RT-PCR, in upper jejunum of *Vil-Cre Tmed9KI$^{mut/mut}$*, *Vil-Cre Tmed9KI$^{wt/mut}$*, and control *Tmed9KI$^{mut/mut}$* (n= 8 for each genotype, respectively). **d** Relative *Tmed9* mRNA levels, measured by qRT-PCR, in indicated tissues of *Vil-Cre Tmed9KI$^{mut/mut}$*,*Vil-Cre Tmed9KI$^{wt/mut}$*, and *Tmed9KI$^{mut/mut}$* control mice (n= 3,3,4 for each genotype). **e** Heatmap of transcripts from mRNAseq encoding solute carriers from isolated enterocytes of *Vil-Cre Tmed9KI$^{mut/mut}$*, and *Tmed9KI$^{mut/mut}$* control mice shown as Log2FC (n= 3,3 per group). **f** Colonization experiment of 10-week-old, pathogen-free *Vil-Cre Tmed9KI$^{mut/mut}$*, and *Tmed9KI$^{mut/mut}$* control mice that were gavaged with 10^8 CFU stationary phase *Salmonella* Typhimurium (strain SL1433) cultures grown overnight. Bacterial levels in spleen, feces and mesenteric lymph nodes (mLNs) were determined by diluted plating of homogenized tissues. Colony formation unit per gram mLN and per mLN, colony formation unit per gram of cecal content, and colony formation unit per gram of spleen (n= 5 per genotype). **g,h** Quantitative analysis of LYZ-positive cells (g), and BrdU-positive cells (h) in *Vil-Cre Tmed9KI$^{mut/mut}$*, *Vil-Cre Tmed9KI$^{wt/mut}$*, and control mice *Tmed9KI$^{mut/mut}$* (n= 4,4,5 respectively per genotype). **i** Immunoblot of Cleaved Caspase 3 protein in extracts of upper jejunum from *Vil-Cre Tmed9KI$^{mut/mut}$* and *Tmed9KI$^{mut/mut}$* control mice. β-ACTIN was used as a loading control. Quantitative analysis of densitometric measurements are shown on the right. Each lane represents a lysate from a different mouse (n= 4 per genotype). **j** Heatmap of transcripts analyzed by RNAseq encoding positive regulators of cell cycle genes (GO 0051726) in the upper jejunum of *Vil-Cre Tmed9KI$^{mut/mut}$* and *Tmed9KI$^{mut/mut}$* control mice shown as Log2FC (n= 3). **k,l** Heatmap of transcripts from RNAseq data encoding antioxidant genes (top) and *Notch* as well as Notch targets (bottom) (k) and antioxidant genes (l) of the upper jejunum from *Vil-Cre Tmed9KI$^{mut/mut}$* and *Tmed9KI$^{mut/mut}$* control mice shown as Log2FC (n=3). Data is plotted as mean ± SD. Statistical significance was evaluated by two-tailed t-test (i), one way Anova with Tukey's correction for multiple comparison (b–d, g,h), or by two tailed Mann-Whitney test (f).

Supplementary Table 1

Antigen	Raised in	Dilution	Producer	Catalog #
Tmed9	Rabbit	1:200	Sigma-Aldrich	HPA014650
Lysozyme-C	Goat	1:200	Santa Cruz	sc-27958
b-catenin	Mouse	1:1000	BD Biosciences	610154
FLAG-M2	Mouse	1:500	Sigma-Aldrich	F1804-200UG
Cleaved-caspase3	Rabbit	1:1000	Cell Signaling	9661S
BrdU	Rat	1:400	Abcam	ab6326
b-Actin	Rabbit	1:1000	Cell Signaling	4970
g-Tubulin	Mouse	1:1000	Sigma-Aldrich	T6557
Glut2	Rabbit	1:500	Santa Cruz	sc-9117
H3	Rabbit	1:1000	Cell Signaling	4499S
Occludin	Rabbit	1:1000	Abcam	ab167161
Claudin1	mouse	1:1000	Thermo Scientific	37-4900
Olfm4	rabbit	1:400	Cell Signaling	39141
