# Supplementary material for: miR-802 regulates Paneth cell function and enterocyte differentiation in the mouse small intestine
Source: Nat Commun. 2021 Jun 7;12:3339. doi: 10.1038/s41467-021-23298-3 (PMC8184787; doi:10.1038/s41467-021-23298-3)
Supplement: Supplementary file 2 — Description of Additional Supplementary Files [file 41467_2021_23298_MOESM2_ESM.pdf]

Title: Supplementary Data 1.

Description: Oligonucleotide primer sequences used for RT-qPCR expression analysis. Sequences are shown in 5’ to 3’ direction.
