# Supplementary material for: miR-802 regulates Paneth cell function and enterocyte differentiation in the mouse small intestine
Source: Nat Commun. 2021 Jun 7;12:3339. doi: 10.1038/s41467-021-23298-3 (PMC8184787; doi:10.1038/s41467-021-23298-3)
Supplement: Supplementary file 4 — Reporting Summary [file 41467_2021_23298_MOESM4_ESM.pdf]

nature research

Corresponding author(s): Stoffel, Markus

Last updated by author(s): 04/03/2021

Reporting Summary

Nature Research wishes to improve the reproducibility of the work that we publish. This form provides structure for consistency and transparency in reporting. For further information on Nature Research policies, see our Editorial Policies and the Editorial Policy Checklist.

Statistics

For all statistical analyses, confirm that the following items are present in the figure legend, table legend, main text, or Methods section.

n/a	Confirmed	
☐	☒	The exact sample size (n) for each experimental group/condition, given as a discrete number and unit of measurement
☐	☒	A statement on whether measurements were taken from distinct samples or whether the same sample was measured repeatedly
☐	☒	The statistical test(s) used AND whether they are one- or two-sided<br>*Only common tests should be described solely by name; describe more complex techniques in the Methods section.*
☒	☐	A description of all covariates tested
☐	☒	A description of any assumptions or corrections, such as tests of normality and adjustment for multiple comparisons
☐	☒	A full description of the statistical parameters including central tendency (e.g. means) or other basic estimates (e.g. regression coefficient) AND variation (e.g. standard deviation) or associated estimates of uncertainty (e.g. confidence intervals)
☐	☒	For null hypothesis testing, the test statistic (e.g. F, t, r) with confidence intervals, effect sizes, degrees of freedom and P value noted<br>*Give P values as exact values whenever suitable.*
☒	☐	For Bayesian analysis, information on the choice of priors and Markov chain Monte Carlo settings
☒	☐	For hierarchical and complex designs, identification of the appropriate level for tests and full reporting of outcomes
☒	☐	Estimates of effect sizes (e.g. Cohen's d, Pearson's r), indicating how they were calculated

Our web collection on statistics for biologists contains articles on many of the points above.

Software and code

Policy information about availability of computer code

Data collection	n.a.
Data analysis	n.a.

For manuscripts utilizing custom algorithms or software that are central to the research but not yet described in published literature, software must be made available to editors and reviewers. We strongly encourage code deposition in a community repository (e.g. GitHub). See the Nature Research guidelines for submitting code & software for further information.

Data

Policy information about availability of data

All manuscripts must include a data availability statement. This statement should provide the following information, where applicable:

- Accession codes, unique identifiers, or web links for publicly available datasets
- A list of figures that have associated raw data
- A description of any restrictions on data availability

Sequencing data have been deposited in ArrayExpress under accession number (pending). The authors declare that the data supporting the findings of this study are available within the paper and its supplementary information files. Data not included are available from the corresponding authors upon reasonable request.

Field-specific reporting

Please select the one below that is the best fit for your research. If you are not sure, read the appropriate sections before making your selection.

- [x] Life sciences
- [ ] Behavioural & social sciences
- [ ] Ecological, evolutionary & environmental sciences

For a reference copy of the document with all sections, see nature.com/documents/nr-reporting-summary-flat.pdf

Life sciences study design

All studies must disclose on these points even when the disclosure is negative.

Sample size	Included in Figure legends
Data exclusions	No data were excluded
Replication	indicated in figure legends
Randomization	Different allelic combinations of littermate mutant/wildtype mice were used
Blinding	Genotype of mice were generally blinded to investigator studying the phenotype

Reporting for specific materials, systems and methods

We require information from authors about some types of materials, experimental systems and methods used in many studies. Here, indicate whether each material, system or method listed is relevant to your study. If you are not sure if a list item applies to your research, read the appropriate section before selecting a response.

Materials & experimental systems

n/a	Involved in the study	
[ ]	[x]	Antibodies
[ ]	[x]	Eukaryotic cell lines
[x]	[ ]	Palaeontology and archaeology
[ ]	[x]	Animals and other organisms
[x]	[ ]	Human research participants
[x]	[ ]	Clinical data
[x]	[ ]	Dual use research of concern

Methods

n/a	Involved in the study	
[x]	[ ]	ChIP-seq
[ ]	[x]	Flow cytometry
[x]	[ ]	MRI-based neuroimaging

Antibodies

Antibodies used	All antibodies are commercial an described
Validation	None, well established Ab

Eukaryotic cell lines

Policy information about cell lines

Cell line source(s)	ATCC
Authentication	New line from ATCC, no authentication
Mycoplasma contamination	Not checked, newly acquired
Commonly misidentified lines (See ICLAC register)	*Name any commonly misidentified cell lines used in the study and provide a rationale for their use.*

Animals and other organisms

Policy information about studies involving animals; ARRIVE guidelines recommended for reporting animal research

Laboratory animals	Genetically modified mice

Wild animals

Provide details on animals observed in or captured in the field; report species, sex and age where possible. Describe how animals were caught and transported and what happened to captive animals after the study (if killed, explain why and describe method; if released, say where and when) OR state that the study did not involve wild animals.

Field-collected samples

For laboratory work with field-collected samples, describe all relevant parameters such as housing, maintenance, temperature, photoperiod and end-of-experiment protocol OR state that the study did not involve samples collected from the field.

Ethics oversight

Kantonale Tierschutzkomission Zurich. Animal licence approval numbers ZH 143/2020 and ZH 043/2019.

Note that full information on the approval of the study protocol must also be provided in the manuscript.

Flow Cytometry

Plots

Confirm that:

- [x] The axis labels state the marker and fluorochrome used (e.g. CD4-FITC).
- [x] The axis scales are clearly visible. Include numbers along axes only for bottom left plot of group (a 'group' is an analysis of identical markers).
- [x] All plots are contour plots with outliers or pseudocolor plots.
- [x] A numerical value for number of cells or percentage (with statistics) is provided.

Methodology

Sample preparation

The crypt fraction isolated from upper jejunum was used to isolate Paneth Cells and Intestinal Stem Cells.

Instrument

BD FACS Aria Fusion sorter.

Software

Data was collected as FCS files and analyzed in FlowJO.

Cell population abundance

Paneth Cells are around 15% of the single, live, negative lineage population as shown in the main figure. To enrich for the Paneth cell population only the SSC-A high fraction (shown in the main figure) was sorted for further use, since it is known from the literature that this fraction contains 95% positive Paneth Cells. The Paneth cell enriched population was further validated by qRT-PCR for the Paneth cell marker (shown in Supplementary Figure).

The Stem cell enriched fraction was sorted as single, live, CD45-, CD24+ LOW. This consist in only an enriche fraction intestinal stem cells fraction, as previously shown in the literature and confirmed by qRT-PCR data in the Supplementary figures.

Gating strategy

Paneth cells gating
Gated: Epcam+, CD24 SSChigh, CD45–, CD31–, Ter119– live cells

Intestinal stem cell population
live single, CD24+, CD45-

- [x] Tick this box to confirm that a figure exemplifying the gating strategy is provided in the Supplementary Information.
